# Endothelial ILK induces cardioprotection by preventing coronary microvascular dysfunction and endothelial-to-mesenchymal transition

**DOI:** 10.1007/s00395-023-00997-0

**Published:** 2023-07-14

**Authors:** P. Reventun, S. Sánchez-Esteban, A. Cook-Calvete, M. Delgado-Marín, C. Roza, S. Jorquera-Ortega, I. Hernandez, L. Tesoro, L. Botana, J. L. Zamorano, C. Zaragoza, M. Saura

**Affiliations:** 1 Facultad Medicina, Depto. Biología Sistemas (UD Fisiología), Universidad de Alcalá, IRYCIS, Mod 2 Planta 0, Ctra Madrid, Barcelona Km 33,500, Alcalá de Henares, Madrid, Spain; 2 Present Address: School of Medicine, Department of Medicine, Cardiology Division, Johns Hopkins University, Baltimore, MD United States; 3 Unidad Mixta de Investigación Cardiovascular, Universidad Francisco de Vitoria, IRYCIS, Pozuelo de Alarcón, Madrid, Spain; 4 Servicio Cardiología, Hospital Universitario Ramón y Cajal, Madrid, Spain; 5 Centro de Investigación Biomédica en Red en Enfermedades Cardiovasculares (CIBERCV), Madrid, Spain

**Keywords:** Integrin-linked kinase, Endothelial to mesenchymal transition, Microvascular dysfunction, Microvessel remodeling, Ischemia, Fibrosis

## Abstract

Endothelial dysfunction is an early event in coronary microvascular disease. Integrin-linked kinase (ILK) prevents endothelial nitric oxide synthase (eNOS) uncoupling and, thus, endothelial dysfunction. However, the specific role of endothelial ILK in cardiac function remains to be fully elucidated. We hypothesised that endothelial ILK plays a crucial role in maintaining coronary microvascular function and contractile performance in the heart. We generated an endothelial cell-specific ILK conditional knock-out mouse (ecILK cKO) and investigated cardiovascular function. Coronary endothelial ILK deletion significantly impaired cardiac function: ejection fraction, fractional shortening and cardiac output decreased, whilst left ventricle diastolic internal diameter decreased and E/A and E/Eʹ ratios increased, indicating not only systolic but also diastolic dysfunction. The functional data correlated with extensive extracellular matrix remodelling and perivascular fibrosis, indicative of adverse cardiac remodelling. Mice with endothelial ILK deletion suffered early ischaemic-like events with ST elevation and transient increases in cardiac troponins, which correlated with fibrotic remodelling. In addition, ecILK cKO mice exhibited many features of coronary microvascular disease: reduced cardiac perfusion, impaired coronary flow reserve and arterial remodelling with patent epicardial coronary arteries. Moreover, endothelial ILK deletion induced a moderate increase in blood pressure, but the antihypertensive drug Losartan did not affect microvascular remodelling whilst only partially ameliorated fibrotic remodelling. The plasma miRNA profile reveals endothelial-to-mesenchymal transition (endMT) as an upregulated pathway in endothelial ILK conditional KO mice. Our results show that endothelial cells in the microvasculature in endothelial ILK conditional KO mice underwent endMT. Moreover, endothelial cells isolated from these mice and ILK-silenced human microvascular endothelial cells underwent endMT, indicating that decreased endothelial ILK contributes directly to this endothelial phenotype shift. Our results identify ILK as a crucial regulator of microvascular endothelial homeostasis. Endothelial ILK prevents microvascular dysfunction and cardiac remodelling, contributing to the maintenance of the endothelial cell phenotype.

**Supplementary Information:**

The online version contains supplementary material available at 10.1007/s00395-023-00997-0.

## Introduction

Despite decades of research, coronary artery disease (CAD)/ischaemic heart disease remains one of the leading causes of cardiac events worldwide [42]. Adverse cardiac remodelling in association with ischaemic heart disease can lead to heart failure. Increased extracellular matrix deposition causes cardiac stiffness and diastolic dysfunction, resulting in cardiomyocyte hypertrophy and left ventricular systolic dysfunction. In addition, perivascular fibrosis may impair oxygen delivery to cardiomyocytes and thus exacerbate myocardial ischaemia. Although most of the efforts in cardioprotective strategies have been focussed on reducing the infarct size, many studies have shown that functional and structural abnormalities of the coronary microcirculation may be involved in both acute and chronic cardiac ischaemic syndromes [15]. In addition, coronary microvascular dysfunction (CMD) contributes to myocardial ischaemia in a significant number of patients with normal or near-normal coronary arteries on angiography. CMD is then associated with a high risk of major adverse cardiovascular events including myocardial infarction, progressive heart failure, stroke and even sudden death [32]. CMD may result from abnormal coronary microvascular dilatory responses, coronary microvascular spasm or extravascular compressive forces [20]; however, treating coronary microvascular dysfunction remains a major challenge since its mechanisms are not well understood.

Integrin Linked Kinase (ILK) is a serine/threonine kinase that binds to the cytoplasmic domain of β-integrins and participates in cardiac remodelling after MI [30, 56]. ILK lies upstream of many intracellular signalling pathways potentially involved in such effect, including angiogenesis, mechanotransduction, extracellular matrix-mediated signalling, cell survival, proliferation, differentiation and apoptosis [26, 29]. The ILK role in cardiomyocyte contractile function is well studied. Mutations in ILK have been reported in human patients with dilated cardiomyopathy (DCM) [23] and targeted ILK deletion in the murine heart causes spontaneous DCM and heart failure [54]. However, the specific role of endothelial ILK in cardiac function is poorly defined.

Endothelial cells play an essential role in cardiac function, especially in the microvasculature, where the closest endothelial–cardiomyocyte interactions occur [2]. In vascular endothelium, ILK expression in endothelial cells plays a critical function in regulating vasomotor tone by protecting eNOS from uncoupling. ILK prevents endothelial dysfunction and the downstream cascade of events, including oxidative stress, hypertension, acute inflammation and atherosclerosis [12]. Moreover, we found that ILK proteolytic degradation in aortic endothelium accelerates atherosclerotic progression [37].

It is described that growth factors, inflammation and hypoxia may induce a phenotypic switch in endothelial cells, termed endothelial‐to‐mesenchymal transition (endMT) [35]. During endMT, endothelial cells lose endothelial-specific proteins (CD 31 and VE-Cadherin) and acquire specific myofibroblast markers (α‐smooth muscle actin [α‐SMA] and vimentin). EndMT can be induced after MI, contributing to cardiac fibrosis [35]. Indeed, defective endMT can attenuate cardiac fibrosis after MI, suggesting the involvement of endMT in the pathology of myocardial fibrosis after ischaemia [18]. Recently, we have demonstrated a link between ILK decreased expression in aortic valve endothelial cells and endMT [39].

To gain an in-depth understanding of the role of endothelial ILK in cardiac protection, we generated an endothelium-specific ILK conditional knock-out mouse to study the role of ILK on the coronary microvascular function and its impact on cardiac structure and contractile performance.

## Materials and methods

A detailed listing of the reagents and antibodies used through the study is provided in supplementary Table 1.

A comprehensive methods section is included in the Data Supplement.

### Animals

To study the in vivo role of endothelial ILK in the heart, we bred mice with two transgenes in the C57BL6 background: (a) the ILK gene, with exons 1 and 2 flanked by *loxP* sites (ILK^lox/lox^) [9]; (b) Cre recombinase fused with a modified oestrogen receptor under control of an EC-specific VE-cadherin promoter (Cdh5-PAC-Cre^ERT2^) [25]. Mice were housed in our animal facilities in isolated rooms**.** All experimental and other scientific procedures with animals conformed to EU Directive 2010/63EU and Recommendation 2007/526/EC, enforced in Spanish law under Real Decreto 53/2013. The local ethics committees and the Animal Protection Area of the Comunidad Autónoma de Madrid (PROEX 231.2/20) and Gobierno de Navarra (115–21.) approved animal protocols.

### Animal protocol study

The mice (10–12 weeks old, ~ 30 g) with ILK ^lox/lox^/Cdh5- PAC-CreERT2 genotype were randomly assigned to either the control group (receiving vehicle (VH; corn oil) and named CT mice afterwards) or tamoxifen group (receiving Tamoxifen (TXF; 1.5 mg/day) to delete endothelial ILK, named ecILK cKO mice). TXF or VH was injected intraperitoneally for five consecutive days. To avoid the influence of oestrogen fluctuations in the female mice, only male mice were used to assure consistence of results in our study. Cardiovascular function parameters were obtained in CT and ecILK cKO mice before treatment (basal), one week (1W), two weeks (2W) and three weeks (3W) after treatment. The efficiency and the specificity of recombination were confirmed by PCR (Suppl. Figure 1A).

Echocardiography, ECG and blood pressure measurements were performed by a single experienced operator in a blinded fashion. Data analyses were blinded throughout all the experimental procedures.

Unless otherwise indicated, mice were sacrificed three weeks after TXF or VH administration. In selected experiments, we performed follow-up and sacrifice at three-day intervals during the first two weeks. Finally, a subset of mice CT and ecILK cKO mice were treated with losartan (0.1 mg/ml) or PBS, administered in the drinking water, at the beginning of TXF and VH administration, as reported previously [40]. Mice were monitored by echocardiogram, ECG and blood pressure measurements and sacrificed at 1, 2 and 3 weeks. A total of 150 mice were used to perform all of these experiments.

Group sizes were determined according to our previous experience with a global inducible ILK cKO mouse model [12]. After we observed that endothelial ILK deletion induced a decline in the left ventricle ejection fraction (EF), the number of mice in each group was adjusted based on power calculations for the primary parameter (EF) with mean differences and standard deviations taken from pilot data at power 90% with a standard level of significance of 0.05. This results in five mice/experimental condition thus, we routinely assigned enough mice per group to have at least five mice per experimental time point.

### Cell cultures

Murine aortic ECs (MAECs) were isolated from VE-Cadh-Cre + /ILK^lox/lox^ mice aortas as previously reported [12]. MAECs were selected by their ability to express the vascular endothelial cadherin (CD144; BD 560411) protein and purified with a flow cytometry cell sorter (FACSAria™ Fusion cell sorter, BD, NJ, USA). Purification was verified by confocal microscopy of MAECs double stained with CD31 antibodies.

Human Cardiac Microvascular Endothelial Cells (ScienCell, Carlsbad, CA, USA) were cultured in Endothelial cell medium (ScienCell, Carlsbad, CA, USA) in gelatin coated plates and supplemented with Endothelial Cell Medium Supplement Kit (Cell Biologist) including: VEGF, Heparin, EGF, FGF, Hydrocortisone, L-Glutamine, Antibiotic–Antimycotic Solution and 10% Foetal Bovine Serum.

### ECG recordings

Mice were anaesthetised with isoflurane (1–1.5%, 1 ml/min oxygen) in an induction chamber to maintain a heart rate of ~ 400 bpm. Fine-needle electrodes (25G) were inserted subcutaneously at the level of both armpits and left groin and connected to an AC amplifier (Cyberamp, Axon Instruments). The ECG leads were placed initially in the lead II configuration and exchanged when required, to the lead I and lead II at the pre-amplifier. The signals were amplified 500 times and band-pass filtered between 1 and 100 Hz, digitised at 1000 Hz (Power 1401, CED, UK) and stored for off-line analysis using Spike 2 software (CED, UK) [52]. In some animals a surface ECG recording was performed (EDAN SE 601; ASMEDIC Spain).

### Echocardiography

Mouse hearts were visualised by echocardiography using a Vivid Q ultrasound system equipped with a 12.5 MHz scan head. Mice were anaesthetised with 1.5% isoflurane gas, resulting in a heart rate of approximately 400 beats/min. Parasternal short-axis-view images of the heart were recorded in a B-mode to allow M-mode recordings by positioning the cursor in the parasternal short-axis view perpendicular to the interventricular septum and posterior wall of the left ventricle [5]. From these recordings, the following parameters were determined using the on-site software cardiac package: systolic and diastolic Interventricular septum thickness (IVS), systolic and diastolic left-ventricle internal diameter (LVID), systolic and diastolic left-ventricle posterior Wall thickness (LVPW), left-ventricle ejection fraction (EF), left ventricle shortening fraction (FS) and heart rate (HR). A comprehensive echocardiographic analysis including diastolic function was performed using a Vevo3100 (Visualsonics, Toronto, Canadá) equipped with an MX550 transducer. The images obtained were measured offline using the cardiac measurement package integrated into the VevoLab software (v5.7.1).

Cardiac function and morphology of the left ventricle (LV) were estimated from the parasternal short-axis view (PSAX) using M-mode at the mid-papillary level. To that end, the M-mode gate was placed perpendicular to the interventricular septum (IVS) and LV walls to derive systolic function parameters [stroke volume (SV), ejection fraction (EF), fractional shortening (FS), and cardiac output (CO)]. LV wall thickness and chamber dimensions [end-systolic volume (ESV), end-diastolic volume (EDV), LV mass, anterior and posterior wall thickness (LVAW, LVPW)] were also estimated from this view. Mitral valve (MV) and tricuspid valve (TV) inflow were assessed from the apical 4 chamber view using pulsed wave Doppler (PWD) mode for estimation of diastolic function. MV measurements performed were as follows: MV early (E) and late (A) atrial contraction waves, E/A ratio, deceleration time (MVDT), isovolumic contraction time (IVCT), isovolumic relaxation time (IVRT) and ejection time (ET). Myocardial performance index (MPI), calculated by [(IVCT + IVRT)/ET], was used to assess global LV systolic function. TV measurements included TV early (TVE) and TV atrial (TVA) wave peaks and the TVE/A ratio. From this view, diastolic annular velocities (E′, A′) were also captured by Tissue Doppler imaging (TDI) at the septal mitral annulus. All measurements were averaged on at least 10 cardiac cycles.

### Measurement of coronary flow reserve

In a subset of mice (5 mice/group), coronary flow reserve was analysed using a high-resolution ultrasound imaging system (Vevo 3100, Visualsonics, Toronto, Canada) equipped with a RMV-710B transducer with a frequency of 25 MHz and a fixed focal length of 15 mm mounted on an integrated rail system as described [38]. Mice were anaesthetised with isoflurane and left anterior descending coronary artery (LAD) flow velocity was measured under a modified four-chamber view. To that aim, Doppler measurements were acquired at baseline coronary flow (1% isoflurane), and under conditions of maximum hyperemic flow (3% isoflurane). Heart rate was monitored whilst the mouse was anaesthetised with 1% (470–520 bpm, non-hyperemic) and 2.5% (250–300 bpm, hyperemic) isoflurane. Non-hyperemic and hyperemic peak diastolic flow velocities were obtained from averaging 3 –5 cardiac cycles, and coronary flow reserve (CFR) was calculated as the ratio of hyperemic to non-hyperemic peak diastolic flow velocity.

### Non‑invasive blood pressure

Non-invasive blood pressure measurements were obtained in conscious animals using a tail-cuff sphygmomanometer (LE 5001 Pressure Meter; Letica Scientific Instruments, Hospitalet, Spain) according to a previously published protocol [40]. The animals were trained for 5 d before starting the measurement to prevent stress and were pre-warmed to 30 °C with a heater. Blood pressure was measured several times between 9:00 and 12:00 AM, and pressure values were considered acceptable at ten consecutive measurements. This method has been proofed in accuracy in comparison with radiotelemetric measurement of implanted catheter devices by Feng et al. [7]. Measurements were preceded by three training sessions to acclimate the animal before the baseline measurement and avoid stress reactions that may affect vascular tone and resting blood pressure.

### Histology

Hearts were excised under 2% isoflurane anaesthesia. KCl (10%) was injected into the LV chamber before the hearts were harvested to arrest them in diastole. Hearts were washed with PBS (ice-cold) and then fixed in a 10% formalin solution, dehydrated in ethanol and then paraffin-embedded as previously described [5]. Tissue Sects. (5 μm) were deparaffinized, rehydrated and stained with Masson's trichrome staining kit (EMD Millipore Corporation, Billerica, Massachusetts) and Sirius red staining (Sigma-Aldrich, San Luis, Missouri) for fibrosis quantification.

For vascular morphometry measurements, cardiac perfusion fixation was performed. The whole heart of each mouse was gravity-perfused with 1 × PBS followed by 4% paraformaldehyde in PBS. Fixed hearts were immersed overnight in 10% formalin and then stored in 70% ethanol for up to 1 day before being processed and embedded in paraffin blocks.

### Morphometric analysis

Myocardial fibrosis quantification was performed in Masson’s Trichrome stained sections. Fibrotic areas were stained in blue. Six non-overlapping fields of the left ventricular myocardium, including free wall and interventricular septum and the right ventricle, were imaged under a Nikon microscope at 10 × magnification, thereby covering the entire heart tissue. Four cross sections per heart were used. The percentage of fibrotic tissue was quantified using the colour‐threshold plugin of ImageJ software (https://imagej.nih.gov/ij/), which measures the blue‐stained area in relation to the total myocardial area. Fibrosis data is presented as the average value of fibrotic area respect to total area of the heart of each mouse and expressed as the percentage of fibrosis.

To evaluate perivascular fibrosis, we used Masson’s trichrome stained myocardial regions with transversely oriented myocytes and circular section arteries with lumen diameter between 10 μm and 100 μm. At least, ten cross sections were analysed for each mouse. Perivascular fibrosis was quantified as the ratio of the fibrosis area surrounding the vessel to the total vessel area using NIS element D3.2 Nikon software (Nikon, USA).

To perform the capillary counting and measure the intercapillary distance, slides were co-stained with FITC-conjugated isolectin B4 (IB4). Hoechst was added to visualise nuclei. For each heart, positive IB4 endothelial cells were manually counted from at least six random high-power fields from two different heart regions using the cell counting tool of ImageJ (ImageJ version 1.5.1, National Institutes of Health, Bethesda, MD) and normalised to area. Capillary density was calculated by dividing the number of capillaries by total area analysed and expressed as capillaries/mm^2^. Intercapillary distance was calculated by analysing the average of 150 intercapillary distances from three fields from each heart region.

The coronary arterial wall thickness was quantified in Masson’s Thrichrome stained sections and confirmed in cardiac sections stained with α-SMA. Arteriolar density quantifications were done in similar fashion as the capillary density quantifications.

The arterioles were classified according to size between 60 and 10, and > 100 μm diameter in the short-axis cross-section. For each slide, six arterioles per size classification were selected at random and analysed for arteriolar wall thickness. Briefly, the total vessel area, lumen area, and vessel diameter and lumen diameter were manually delineated using ImageJ (ImageJ version 1.6.0, National Institutes of Health Bethesda, MD). For the analysis of microvascular remodelling, we analysed the wall thickness of each arteriole (intima and media) averaged from measurements made at four different points around the vascular wall. Results are displayed as the ratio of vessel wall thickness to the lumen area for the two categories of arteriolar size described above.

To evaluate the extent of cardiomyocyte hypertrophy, cross-sectional images of cardiomyocytes were incubated with FITC–conjugated wheat germ agglutinin (WGA) for 1 h. Hoechst was added to visualise the nuclei. Images of six different heart regions were obtained by confocal microscopy (Leica TCS SP5). At least six random high-power fields from cardiomyocytes were used for the quantification of cardiomyocytes area and analysed with Image J software (https://imagej.nih.gov/ij/). The results were presented as cardiomyocyte cross-sectional area expressed in µm^2^ per field for each heart region analysed.

All morphometric measurements were performed in a blindly fashion.

### Coronary microvascular perfusion assay

*Fluorescein isothiocyanate (FITC)-dextran* 150 kDa: 0.2 ml of a 12.5 mg/ml solution, (Sigma, USA) was administered intravenously and circulated for 1 min before sacrifice mice [57]. The hearts were harvested, and cross sections were obtained in a cryostat (8 serial, 60 μm sections/heart). Sections were fixed with paraformaldehyde. After 5 min in ethanol, nuclei were stain with Hoechst. Samples were observed using a Leica TCS SP5 confocal microscope.

*Thioflavins S:* A 4% Thioflavin S dye (Sigma, T1892, USA) was injected into the femoral vein 1 ml/kg and circulated for 1 min before sacrifice. After 1 min of circulation, the hearts were quickly removed and frozen at −20 °C for 20 min, and then horizontally sliced at 1–2 mm thickness. Imaged results were obtained immediately from heart slices placed in a 365 nm ultraviolet light box.

### Necrotic area analysis

In some animals, the heart ischaemia was measured by direct tri-phenyl tetrazolium chloride (TCC) staining [5]. In brief, the hearts were excised, washed with ice-cold PBS, frozen at −20 °C for 20 min, and then transversely cut across the left ventricles. The slices (5 per heart, approximately 3 mm thick) were incubated in 1% TTC solution (pH 7.4, Sigma-Aldrich) at 37 °C for 30 min. The viable tissue was stained red, but the necrotic areas cannot be stained, and thus, appeared pale white. Then, the sections were photographed.

### High-sensitive troponin I (hsTnI) levels

Plasma was diluted 1:4 with PBS, and hsTnI levels were determined by Mouse cardiac troponin I (cTn-I) ELISA kit (CSB-E08421m) from Cusabio Technology LLC (Houston, TX, USA).

### Flow cytometry

MAEC plated in p100 dishes were trypsinized, washed with PBS, and centrifuged at 1500 rpm for 5 min. Cells were fixed and permeabilized with 100% ethanol for 24–36 h. For double labelling experiments, cells were blocked with 3% BSA and incubated for 30 min at 4°C with primary anti-ILK and anti-CD31 or anti-ILK and anti-α-smooth muscle actin primary antibodies. After washing, cells were incubated with the appropriated secondary antibodies conjugated to Alexa Fluor 488 and Alexa Fluor 647. For flow cytometry, 10^6^ cells were used. Data acquisition was performed on a MACSQuant 10 flow cytometer (Miltenyi Biotec) and the data were analysed with the MACSQuant software.

After pre-selection of the endothelial cell population inside scatter (SSC) *versus* forward scatter (FSC) dot plot to exclude debris and doublets, we used the following channels: CD31 (FITC-B1) with ILK (APC-B3) and αSMA (FITC-B1) with ILK (APC-B3). Compensation was not required. Endothelial cells transitioning to mesenchymal cells were identified as ILK^−^/CD31^−^ and ILK^−^/αSMA^+^. We also took into consideration the percentage of cells whose phenotype was CD31^−^ or αSMA^+^. All the results experiments were performed in duplicate and repeated at least three times. Data were expressed as cell percentage respect MAEC gated.

### Statistical analysis

For animal studies, values refer to the number of individual animals used. Statistical significance between 2 groups was examined using unpaired Student’s *t* test. Nonparametric Mann–Whitney tests were used when normality test was not passed and/or sample size was < 4. Comparison of ≥ 3 groups was performed by 1‐way ANOVA followed by Newman–Keuls post hoc multiple comparisons tests. Statistical analysis was performed using Graph Pad Prism v 7.0 software. For western blot and mRNA analysis, every experimental condition was duplicated within each experiment, and each experiment was repeated at least three times. Results are expressed as mean ± SD, and differences were considered statistically significant at *p* < 0.05.

## Results

### Endothelial disruption of ILK causes progressive heart disease

**Fig. 1:**
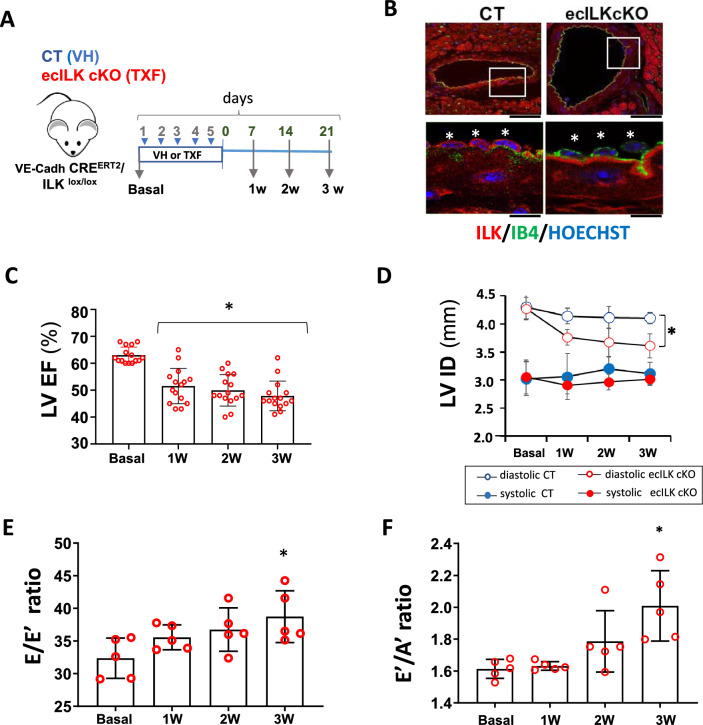
ILK deletion in coronary endothelium leads to myocardial dysfunction. **A** Schematic figure showing the experimental design. VE-Cadh-CreERT2/ILKlox/lox mice were treated (i.p.) during 5 days with Tamoxifen;TXF resulting in endothelial ILK deletion (ecILK cKO in red) or vehicle;VH (CT in blue). Cardiovascular function was tested before treatment (basal) and once a week up to three weeks. Hearts were harvested at 1, 2 and 3 weeks. **B** Representative confocal images of a coronary artery in CT (left panel) and ecILK cKO mice at three weeks stained with anti-ILK (red), endothelial cells were stained with IB4 (green) and nuclei were counterstained in blue with Hoechst. Asterisks in magnification indicate individual endothelial cells showing no expression of ILK (red) in the tamoxifen treated group. Scale bar (upper panel): 25 mm and 10 μm lower panel. **C** Bar graph showing the ejection fraction of the left ventricle (LV EF) in ecILK cKO mice (n = 15, *p < 0.001 vs Basal). **D** Changes in the internal diameter of left ventricle in diastole (LVIDd) and systole (LVIDs) in CT and ecILK cKO mice. (*n* = 15, *p < 0.005 vs CT diastole at three weeks). E. E/Eʹ ratio (LV end–diastolic filling pressure) and **F** Eʹ/Aʹ ratio from ecILKcKO mice (*n* = 5, *p < 0.001 vs Basal). All p values were calculated using ANOVA

An outline of the experimental design is presented in Fig. 1A. Disruption of ILK expression occurred at the endothelial layer of the aorta and coronary arteries, as assessed by confocal microscopy (Fig. 1B and Suppl. Figure 1B). ILK levels did not change in cardiomyocytes nor cardiac fibroblast, confirming endothelial cell-specific deletion of ILK in the ecILK cKO mouse model (Suppl. Figure 1C)*.* Structurally, parental mice hearts (Cadh5-PAC–CRE and ILK lox) appear normal (Suppl. Figure 1D).

Left ventricular (LV) function was assayed in ecILK cKO mice and CT littermates by cardiac ultrasound at baseline and one, two and three weeks after injection of TXF or Vehicle (CT). No differences were observed at baseline. However, ecILK cKO mice exhibited significantly reduced contractility compared to CT mice, as assessed by the ejection fraction measurements (LV EF) (Fig. 1C) and the fractional shortening (LV FS) (Suppl. Figure 2A). This reduction started the first week after deletion but surprisingly did not worsen over time whilst CT mice did not show any significant variation in either EF or FS (Suppl. Figure 2B). Accordingly, cardiac output also decreased, and there was an increase in the left ventricle mass (Suppl. Figure 2C and 2D). End Diastolic Left Ventricle internal diameter (LVID) decreased in ecILK cKO mice compared to CT (Fig. 1D) suggesting diastolic dysfunction as well. Thus, in a separate subset of mice, we measured the E/Eʹ ratio, which represents LV end-diastolic filling pressure (Fig. 1E), the ratio between early (E) and late (atrial-A) ventricular filling velocity (E/A ratio) (Fig. 1F) and the myocardial performance index or TEI, as a measurement of diastolic dysfunction (Suppl. 2E). E/Eʹ and E/A ratios increased over time, and accordingly, the myocardial performance index/TEI, which uses both systolic and diastolic time intervals to assess the global LV cardiac dysfunction, progressively increased over time, becoming significant after three weeks [46]. Thus, ILK deletion leads to significant cardiac systolic and also diastolic dysfunction, eventually leading to HF.

**Fig. 2:**
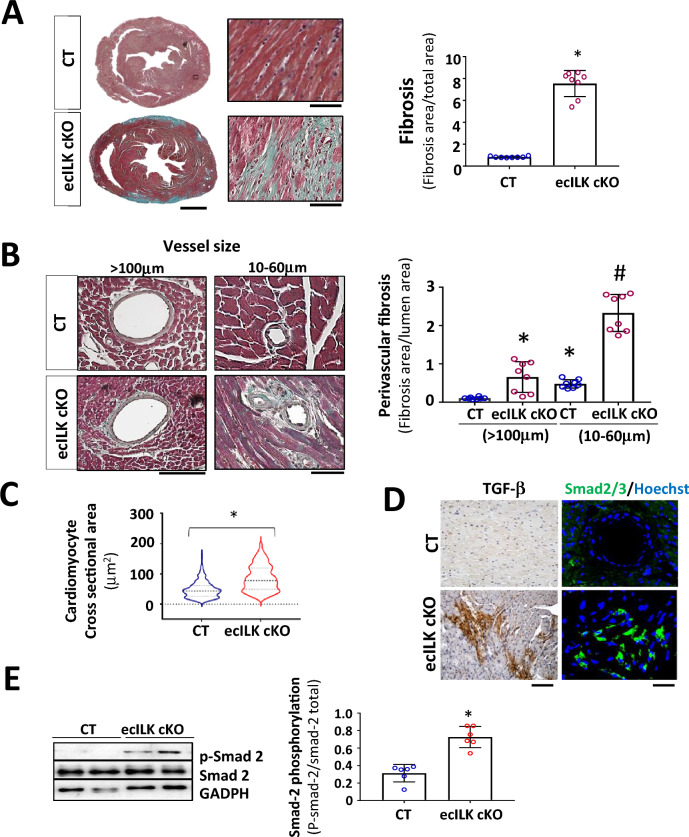
Endothelial-specific ILK deletion leads to adverse remodelling. **A** Representative Masson Trichrome staining of CT and ecILK cKO mice heart sections (Scale bar = 1 mm) showing cardiac lesions three weeks after deletion. Right: Magnification of ecILK cKO and CT hearts. Scale bar: 50 μm. Right panel: quantitative analysis of cardiac fibrosis. (*n* = 8,*p < 0, 005 vs CT). **B** Representative photo-micrographs from ecILK cKO and CT hearts stained with Masson Trichrome showing perivascular fibrosis in myocardial arteries > 100 μm diameter and small arterioles (60–10 μm). Scale bar: 100 μm (left panel); 50 μm (right panel). Right, quantitative analysis of perivascular fibrosis in vessels diameter > 100 μm and small arterioles (60–10 μm). (*n* = 8,*p < 0.001 vs CT > 100 μm; #*p* < 0, 005 vs CT small vessels). **C** Quantitative data of cardiomyocyte (CM) cell surface area; *n* = 6–10 hearts per group with 300–600 CMs analysed per heart. CM area is expressed in μm2. *p < 0, 05 CT vs ecILK cKO. **D** Representative heart sections of CT and ecILK cKO mice staining of TGF-β1 and SMAD2/3 (*n* = 8). Scale bar: 50 μm left and 25 μm right. **E** Immunoblot detection of phosphorylated Smad-2 in total heart lysates from CT and ecILK cKO mice. GAPDH was used as a loading control. Densitometric analysis (right panel) shows a significant increase in the expression of p-Smad2 in ecILK cKO mice (*n* = 6, *p < 0.05 vs CT). *p* values were calculated using Student’s *t *test and one-way ANOVA in **B**

Histological analysis showed extensive areas of replacement fibrosis that, in a more profound analysis, consists in granulation tissue accompanied by interstitial fibrosis (Fig. 2A). As shown in Fig. 2B, there was abundant perivascular fibrosis, mostly around small intra-myocardial coronary arteries (10 to 60 μm) in ecILK cKO after three weeks. In contrast, epicardial arteries only showed a moderate increase in perivascular fibrosis without evident structural alterations. Moreover, cross-sectional area measurements in wheat germ agglutinin-stained heart sections showed cardiomyocyte hypertrophy after three weeks of ILK removal, confirming the echocardiographic data (Fig. 2C).

As detected by picrosirius red staining, total collagen was elevated in mice three weeks after endothelial ILK deletion (Suppl. Fig. 3A). In addition, collagen type I and III expression increased three weeks after deletion indicative of adverse remodelling (Suppl. Figure 3A-C). This pattern is accompanied by an enhancement in pro-fibrotic markers and Extracellular matrix (ECM) expression. As shown in Fig. 2D and E, Transforming growth factor-β (TGF-beta)/Smad-2 pathway was activated in the heart of ecILK cKO mice compared to CT mice. Moreover, CTGF (Connective tissue growth factor) and metalloproteinases (MMP) –2, −9 and −13 showed moderate to significantly increased expression in ecILK cKO mice, as shown in Suppl. Figure 3D-F.

Thus, cardiac dysfunction induced by endothelial ILK deletion is accompanied by an enhancement of ECM remodelling and heart fibrosis.

### Conditional endothelial ILK deletion leads to ischaemic-induced cardiac remodelling

**Fig. 3:**
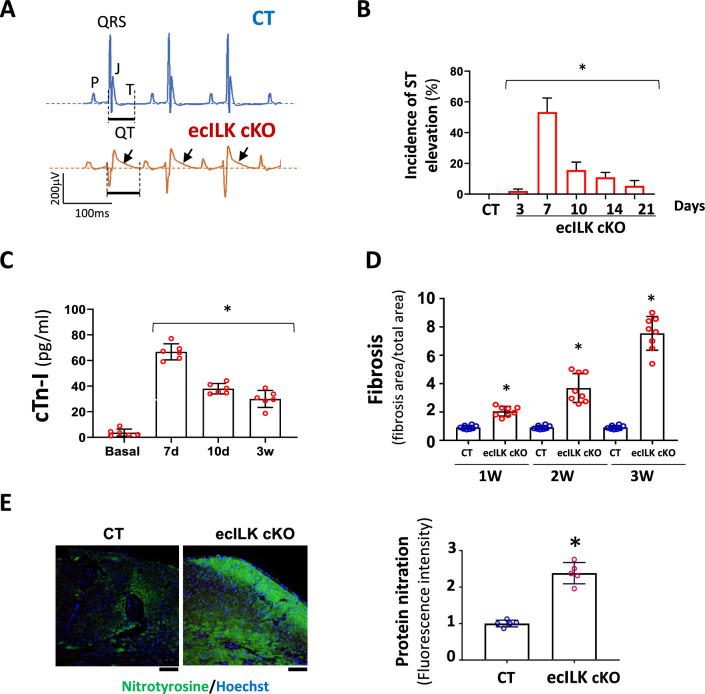
ILK deletion in coronary endothelium promotes myocardial ischaemia. **A** Representative ECG recording (lead II) of CT (upper panel) and ecILK cKO mice (lower panel) 1 week after treatment, showing ST elevation (arrow) in ecILK cKO mice. **B** Incidence of ST elevation in CT (21d) and ecILK cKO mice at different time points post-deletion in six different experimental groups (5–8 mice/group;*p < 0,001 vs CT). **C** Plasmatic cardiac troponin quantitation showing a significant increase 7 days after treatment when compared to basal conditions and remain elevated up to three weeks after deletion (*n* = 6 mice per condition; *p < 0.001 vs Basal). **D** Myocardial Fibrosis from CT and ecILK cKO mice 1, 2 and 3 weeks after deletion/treatment (*n* = 8 mice per condition) *p < 0.05 vs CT at the same time point. **E** Representative heart sections from CT and ecILK cKO mice 3 weeks after deletion stained with nitrotyrosine (green), nuclei were counterstained with Hoetchst (scale bar: 50 μm). Right panel, quantitative analysis showed a significant increase in protein nitration expressed as relative fluorescence intensity in ecILK cKO mice (*n* = 5, *p < 0.05). *p* values were calculated using one-way ANOVA and Student’s *t* test in (**E**)

To investigate the cause of the fibrosis and decreased cardiac function, we also examined cardiac electrical activity. Interestingly, most ecILK cKO mice suffered transient ST-segment elevation within the first 7 to 10 days after treatment. **(**Fig. 3A, B and Suppl. Figure 4A). Plasmatic elevation of cardiac troponins peaked seven days after ILK deletion, suggesting an ischaemic episode (Fig. 3C). Moreover, cardiac fibrosis increased at two and three weeks after tamoxifen administration, suggesting that cardiac remodelling may be the result of the ischaemic-like events (Fig. 3D). Remarkably, we observed increased oxidative stress as detected by the increase in 4-Hidroxinonenal (4-HNE) (Suppl. Figure 3B) and protein carbonylation (Suppl. Figure 3C). Moreover, extensive tyrosine nitration could be observed in ecILK cKO mice at three weeks, which is a hallmark of eNOS uncoupling [12] (Fig. 3E). Together, these results point to endothelial ILK expression as a protective factor from cardiac ischaemia.

**Fig. 4:**
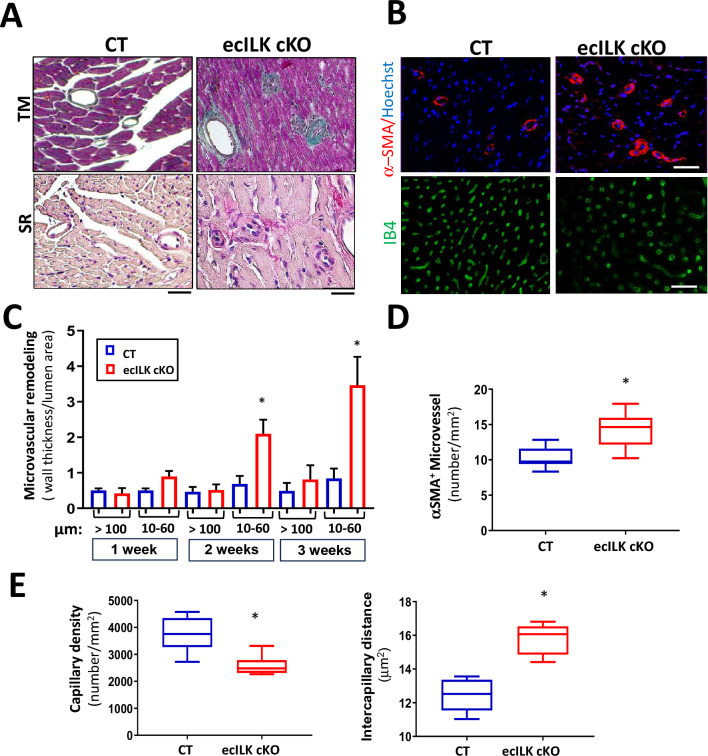
ILK endothelial deletion leads to altered microvascular structure and density. **A** Representative photomicrographs of heart sections stained with Masson Trichrome (upper panel) and Sirius Red (lower panel) showing the remodelled microvasculature in ecILK cKO (right panel) as compared to CT mice (left panel). Scale bar = 25 μm. **B** Representative photomicrographs of heart sections obtained from CT and ecILK cKO mice showing (upper panel) arterioles stained for smooth muscle actin (α-SMA, red) and counterstained in blue with Hoechst and (lower panel) capillaries stained for IB4 (green) Scale bar: 100 mm (*n* = 8). **C** Arteriolar wall thickness-to-lumen area ratio was significantly increased in ecILK cKO mice 2- and 3-weeks post-deletion only in small vessels (10–60 mm) and remained unchanged in large vessels (> 100 mm) (*n* = 8, *p < 0.05 vs CT). **D** Total number of α-SMA-positive microvessels (10-60 μm) was significantly increased in ecILK cKO mice three weeks after deletion (*n* = 6, *p < 0.05 vs CT). **E** In ecILK cKO mice, a significant decrease in capillary density (left panel) together with a significant increase in intercapillary distance (right panel) was observed 3 weeks after treatment when compared to CT mice (*n* = 6, *p < 0.05 vs CT). *p* values were calculated using Student’s *t *test and one-way ANOVA in (**C**)

Coronary atherosclerosis is the leading cause of ischaemic heart disease [31], but ecILK cKO epicardial coronary arteries showed no sign of remodelling. In contrast, small arterioles (10–60 μm) showed evident wall remodelling (Fig. 4A), peaking at three weeks after endothelial ILK deletion (Fig. 4B and C). Indeed, the number of micro-vessels α-SMA-positive showing remodelling was significantly higher in ecILK cKO than in CT mice (Fig. 4D). Cardiac vascularization in ecILK cKO hearts was significantly reduced, as detected by fewer IB4 positive capillaries and the distance between capillaries increased (Fig. 4E).

**Fig. 5:**
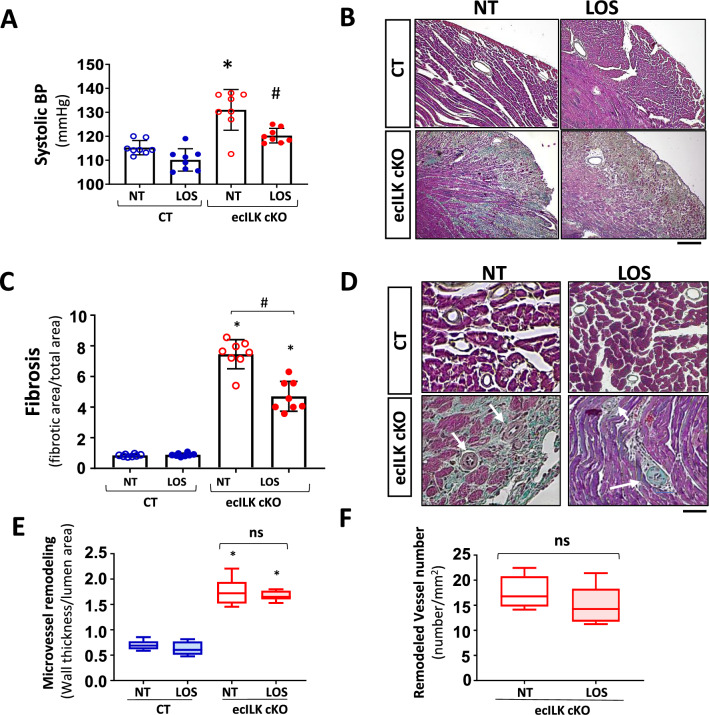
Effect of the antihypertensive drug Losartan, on the myocardial and microvascular remodelling of ecILK cKO mice. CT and ecILK cKO mice were treated with Losartan (LOS, *n* = 8) or left untreated (NT, *n* = 8). **A** Three weeks after treatment, systolic arterial blood pressure was significantly increased in untreated ecILKcKO as compared to CT mice; this increase was prevented by LOS (*p < 0.05 vs CT; # *p* < 0.05 vs NT ecILK cKO). **B** Representative photomicrographs of heart sections obtained from CT and ecILK cKO mice treated as in A, stained with Masson Trichrome showing myocardial fibrosis at three weeks (n = 8) Scale bar: 100 μm. **C** Quantitative analysis of myocardial fibrosis at 3 weeks showing partial prevention in LOS treated vs NT treated ecILKcKO mice (*n* = 8, *p < 0.05 CT vs ecILK cKO; #*p* < 0.01 vs NT ecILK cKO). **D** Representative photomicrographs of heart sections obtained from CT and ecILK cKO mice treated as in A, stained with Masson Trichrome showing remodelled microvasculature (*n* = 8, scale bars: 25 μm, white arrows mark the remodelled vessels). **E** In ecILK cKO mice, the increased arteriolar wall thickness-to-lumen area ratio (in vessels between 10 and 60 mm of diameter) observed three weeks after deletion was unaffected by LOS (*p < 0.001 vs CT, *n* = 8). **F** The increased number of remodelled microvessels in 3 weeks ecILKcKO mice was unaffected by LOS (*p < 0.001 vs CT, *n* = 8). Differences amongst treatment groups were assessed by ANOVA and Student’s *t *test (**F**)

Since ILK deletion can lead to endothelial dysfunction and to study whether the cardiac remodelling results from a hypertensive response we measure systolic and diastolic blood pressure (BP) in CT and ecILK cKO mice at different time points. There was no difference in systolic or diastolic BP within the first week after ILK deletion, whereas at week 3 after treatment, a significant increase in systolic BP was detected (Suppl. Figure 5A and B). To test whether cardiac remodelling was related to increased BP, mice were treated with the antihypertensive drug Losartan (an antagonist to the Angiotensin II receptor). Losartan treatment completely blocked BP increase (Fig. 5A) whilst it only partially inhibited the enhanced fibrosis observed in ecILK cKO mice (Fig. 5B, 5C and Suppl. Figure 5E), and it did not affect the EF and FS (Suppl. Figure 5C and D). Moreover, Losartan had no effect in the microvascular remodelling and did not decrease the number of remodelled vessels (Fig. 5D-F). These results suggested that the microvascular remodelling was not dependent on increased BP.

### Endothelial ILK deletion causes microvascular injury

**Fig. 6:**
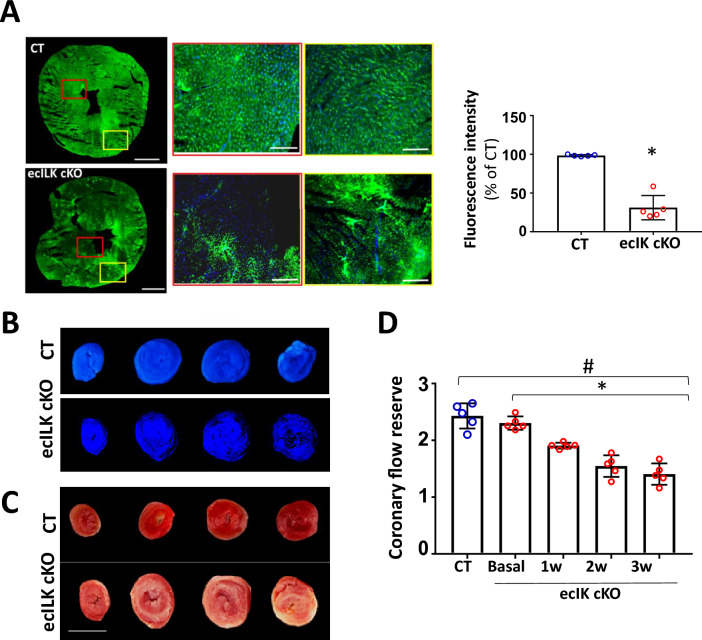
ILK deletion from coronary endothelium promoted microvascular dysfunction. **A** Representative confocal microscopy image of cardiac sections following in vivo dextran-fluorescein isothiocyanate (FITC) injection at 2 weeks after endothelial ILK deletion showing the absence of dextran-FITC (red box) and dextran-FITC accumulation (yellow box) in ecILK cKO microvessels compared to CT mice. Scale bar: 1 mm. Scale bar at magnifications: 100 mm. Right: fluorescence intensity quantitation expressed as percentage of CT (*n* = 6, * *p* < 0.005 vs CT). **B** and **C** Images show Thioflavin S and TTC staining in CT and ecILK cKO mice heart slices three weeks post deletion. **B** Under ultra-violet light, non-fluorescent regions on the Thioflavin S stain highlight areas of compromised coronary blood flow. (*n* = 4). **C** TTC sections indicate region of necrosis appearing white in the ecILK cKO group; CT group did no show infarction (*n* = 4). Scale bar = 5 mm. **D** Coronary flow reserve assessed by echocardiography at baseline and hyperemia in CT (3W) and ecILK cKO mice before (Basal) and at different time points after deletion showing decreased CFR at three weeks compared to CT and Basal (*n* = 5; *p < 0.001 vs Basal; #*p* < 0.001 vs CT). *p* values groups were assessed by ANOVA

Fluorescent myocardial perfusion studies with Dextran-FITC demonstrated perfusion defects as detected by immunofluorescence in ecILK cKO mice (Fig. 6A). Fluorescence was uniform throughout the myocardium of CT mice; a dense and regular network of vessels could be observed in well-perfused apex areas, indicating normal flow (both micro- and macro-vessels). However, endothelial ILK deletion reduced myocardial perfusion based on the distribution of fluorescence areas. After three weeks deletion, ecILK cKO hearts exhibited large dark areas, indicating hypo-perfusion, whilst the fluorescent dye accumulated in the epicardial arteries. Accordingly, an apparent perfusion defect of thioflavin S distribution was observed in the hearts of ecILK cKO mice (Fig. 6B), suggesting an impaired microvascular perfusion, which was coincident with the areas of necrosis as observed in TTC stained hearts sections (Fig. 6C). Together these results suggest that endothelial ILK prevents microvascular damage.

Finally, coronary flow reserve (CFR) was measured as a determinant of coronary microvascular function (Fig. 6D). At 1 week, CFR was significantly attenuated in ecILK cKO mice compared with basal, decreasing further at two- and three weeks post-TXF. No decrease in CFR was observed in CT mice at three weeks.

### Endothelial ILK deletion promotes endothelial-to-mesenchymal transition

To gain insight into the molecular mechanism involved in coronary microvascular dysfunction observed in ecILK cKO mice, we analysed the expression profiles of the circulating miRNAs in CT and ecILK cKO mice. A total of fourteen differentially expressed miRNAs between the CT and ecILK cKO mice were identified; *p* < 0.05 and false discovery rate (FDR) of < 0.05 **(**suppl Fig. 6A). In Suppl. Figure 6B are listed the upregulated and downregulated miRNAs encountered. Using the Ingenuity Pathway analysis (see methods for details), we predicted the activation of several pathways and six of them were related to epithelial-to-mesenchymal transition as significantly activated (Suppl. Figure 6C). Epithelial-to-mesenchymal transition share standard features with endothelial-to-mesenchymal transition [24]. Since the repair response to cardiac injury can induce the transition from endothelial-to-mesenchymal cells and this process may contribute to cardiac fibrosis and arterial remodelling [24], we focus on those pathways.

**Fig. 7:**
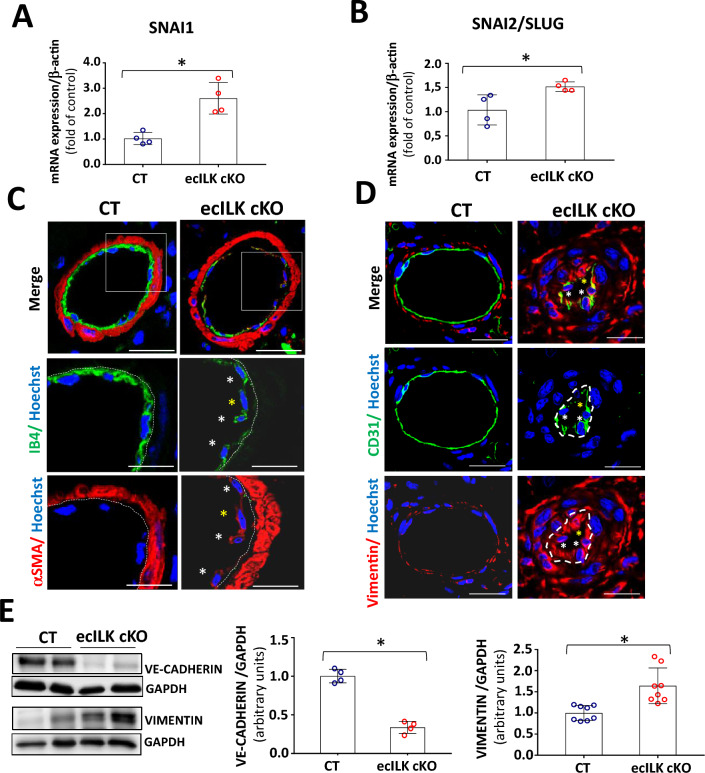
Endothelial ILK deletion induces endMT in coronary microvasculature. RT-qPCR of CT and ecILK cKO mice cardiac extracts showing mRNA expression of endMT transcription factors **A** Snai1 and **B** Snai2 (Slug) are significantly increased in ecILKcKO mice (*p < 0.05, *n* = 4). **C** Representative confocal photomicrographs showing arterioles in cardiac sections from CT and ecILK cKO mice immunostained for IB4 (green) and α-SMA (red). Nuclei were counterstained with Hoechst (blue). White asterisks mark endothelial cells expressing both endothelial and mesenchymal cell markers. Yellow asterisk mark an endothelial cell expressing mostly α-SMA. (Scale bar: 25 mm, *n* = 6). **D** Representative confocal photomicrographs showing small arterioles from CT and ecILK cKO mice immunostained for CD31 (green) and Vimentin (red). Nuclei were counterstained with Hoechst (blue). (Scale bar: 10 μm, *n* = 6). **E** Western blot analysis of VE-cadherin and Vimentin protein expression in CT and ecILK cKO cardiac protein extracts. GAPDH was used as a loading control. Left panel shows representative blots. Quantitative analysis shows a significant decrease of both proteins in ecILK cKO mice (*p < 0.05, *n* = 4–8 mice). p values were calculated using Student’s t test

First, we analysed the mRNA expression of two transcription factors, drivers of endMT Snai1 and Snai2 (Slug), finding that both were upregulated in ecILK cKO mice hearts (Fig. 7A and B**)**. Next, we explored the expression of the endothelial cell markers, CD31, VE-Cadherin and IB4 and myofibroblast markers, vimentin and αSMA in CT and three weeks ecILK cKO mice by confocal microscopy and western blot. As shown in Fig. 7C and D, endothelial cells of ecILK cKO mice exhibited a decreased expression of CD31 and IB4 and increased α-SMA and vimentin in small coronary arteries of ecILK cKO compared to CT mice. Conversely, VE-Cadherin protein expression was downregulated, whereas Vimentin expression was upregulated compared to CT (Fig. 7E).

**Fig. 8:**
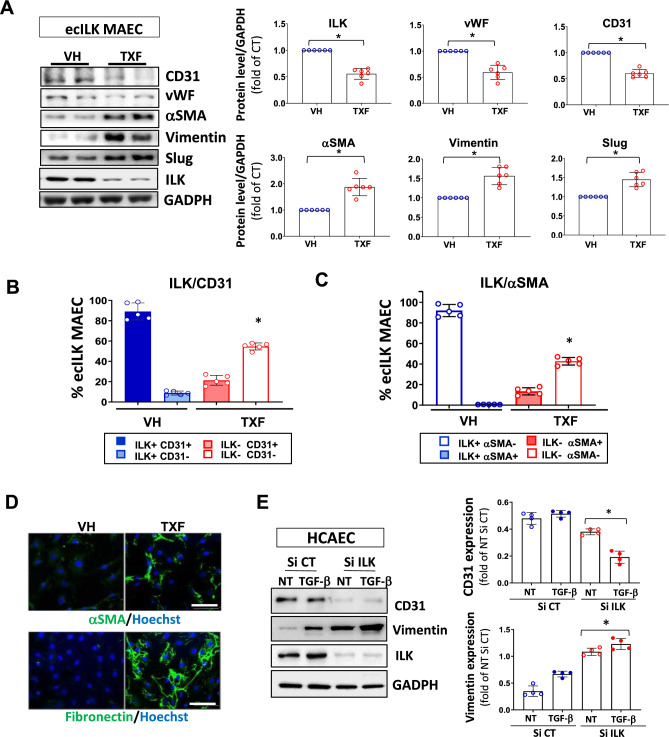
ILK deletion triggers endMT in ecILK MAEC and human coronary endothelial cells. **A** Western blot detection of endothelial (CD31 and vWF) and mesenchymal markers (αSMA and vimentin) expression, Slug and ILK in mouse aortic endothelial cells isolated from Cadh5-PAC-CRE ERT2/ILK flox mice (ecILK MAEC). ILK deletion was induced by treating the cells with Tamoxifen (10^—6^ M) (TXF) or vehicle (VH) for three days. GAPDH protein expression was used as loading control. In the right panel the quantitative analysis shows a significant decrease of ILK, vWF and CD31 in TXF ecILK MAEC as compared to VH (*p < 0.05, *n* = 6) and a significant increase in a-SMA, Vimentin and Slug (*p < 0.05, *n* = 6). Flow cytometry analysis of **B** endothelial marker CD31 and ILK and **C** ILK and α-SMA in VH and TXF treated ecILK MAEC. Figures represent the percentage of cells positive to each marker. VH-treated endothelial cells express CD31 and no α-SMA, whilst half of the TXF treated ecILK cells lose CD31 expression (ILK^-^,CD31^-^) whilst they become positive for α-SMA (ILK^-^, α-SMA +) (*n* = 5). *p < 0.01 vs CT. **D** Representative confocal microphotographs from VH and TXF ecILK MAEC stained for α-SMA (upper panel) or Fibronectin (lower panel). Nuclei were counterstained with Hoechst (blue). Scale bar: 10 μm. **E** Human coronary endothelial cells (HCAEC) were transfected with ILK-specific siRNA (si-ILK) or non-silencing siRNA scramble (si CT) and then, treated with 2 ng/ml TGFβ for 3 days. NT: non-treated. Left panel shows a representative immunoblot for the endothelial cell marker, CD31 and the mesenchymal cell marker, vimentin. GADPH protein expression is used as a loading control. Quantitative analysis (right panel) shows a significant reduction in CD31 expression when ILK was silenced which was further decreased after TGF-β treatment. Vimentin expression was increased in si-ILK cells, and further enhance after TGF- b treatment (*p < 0.05, *n* = 4) Blue circles: SiRNA CT/Red circles: SiRNA ILK. Empty: non-treated, filled: TGF-β treated.*p < 0.05 vs SiCT. *p* values were calculated using ANOVA

To study if ILK deletion can cause endMT, we used Mouse Aortic Endothelial cells (MAEC) isolated from Cadh5-PAC-CRE^ERT2^/ILK^lox^ mice (ecILK-MAEC). Decreased ILK levels in ecILK-MAEC induced by Tamoxifen treatment (TXF) resulted in a marked decrease in the endothelial cell markers, CD31 and von Willebrand (vWF) three days after ILK deletion. At the same time, α-SMA, vimentin, Slug and fibronectin proteins increased compared to vehicle-treated cells (VH) (Fig. 8A and B). To investigate the number of endothelial cells undergoing this phenotypic change, we labelled MAECs isolated from the murine model with antibodies to CD31 and ILK, or α-SMA and ILK, and quantitated them by flow cytometry. In VH-treated cells, nearly all the cells expressed CD31 and ILK; after TXF treatment, 63% of the cells became negative for ILK. Notably, of those, only a reduced fraction still retained the expression of the endothelial cell marker (ILK^**−**^/CD31^**+**^), and the cell population negative for both, ILK and CD31 (ILK^**−**^/CD31^−^) was predominant (Fig. 8B). Accordingly, VH-treated cells expressed ILK but were negative for αSMA; however, after ILK deletion, the number of ILK^−^/αSMA^+^ cells increased (Fig. 8C). Thus, ILK deletion increased the endothelial cell population acquiring a mesenchymal cell marker. Indeed, ILK^−^/αSMA^+^ cells expressed fibronectin abundantly, confirming the tendency towards a fibrotic phenotype (Fig. 8D). Interestingly, roughly 20% of cells retained CD31 and expressed αSMA after ILK deletion, as detected both in culture (Fig. 8B and C) and in stenosed micro-vessels (Fig. 7D), indicating a partial transition.

We extended our experiments to human coronary endothelial cells (HCAEC). ILK-silenced HCAEC showed significantly decreased levels of VE-cadherin whilst inducing vimentin expression. Incubation for three days with transforming growth factor-beta (TGF-β), the main driver of endMT, enhanced endMT transition (Fig. 8E).

These results indicate that ILK activates the endMT programme, which may contribute to coronary microvasculature remodelling and cardiac fibrosis.

## Discussion

Our study shows four significant findings. First, we demonstrate that endothelial ILK expression is necessary to maintain optimal cardiac function. Second, endothelial ILK deletion renders mice vulnerable to ischaemia. Third, endothelial ILK prevents endothelial cell differentiation into mesenchymal cells, thus preventing microvascular remodelling and cardiac fibrosis. Finally, our study points to endothelial ILK as a cardioprotective therapeutic target, highlighting the importance of maintaining normal levels of coronary endothelial ILK expression to preserve microvascular endothelial function.

Conditional ILK deletion in coronary endothelium leads to cardiac contractile impairment, which began one week after ILK deletion. Ejection fraction and, in general, systolic dysfunction showed a sharp decline in the first week but these values stabilise and did not decline further. Moreover, at three weeks, diastolic dysfunction is also evident indicating severe cardiac dysfunction. Several laboratories have studied the role of ILK as a cardioprotective protein. Transgenic mice with cardiac-specific overexpression of *ILKR211A*, a gain-of-function variant of *ILK*, exhibited increased basal LV global systolic and diastolic functions through a SERCA-2a/PLN mechanism [48]. Of note, our mice model exhibited normal levels of myocardial ILK expression. In addition, a number of preclinical studies reported the effect of ILK-MSCs on myocardial infarction using mouse, rat and porcine animal models, which evidenced a sizable reduction in infarct size and fibrosis, and significant improvement in regional perfusion and vessel density when compared with vector-MSCs [28, 30, 56]. It is possible to speculate that ILK-MSC used in preclinical models to reduce infarct size may also differentiate into ECs, restoring ILK levels in coronary endothelium.

Mice showed early ECG abnormalities paralleled by transitory troponin elevation, increased necrosis and myocardial oxidative and nitrosative stress, which strongly suggest myocardial ischaemia. Supporting this data, mouse hearts showed extensive fibrosis, with the appearance of granulation tissue and abundant perivascular fibrosis especially around coronary micro-vessels suggesting a reparative process after myocardial injury. The leading cause of ischaemia is coronary atherosclerotic disease and we have shown that ILK degradation in arterial endothelium induced during inflammatory conditions promotes atherosclerosis progression [37]. However, in the ecILK cKO model, the epicardial coronary arteries did not show any remodelling indicative of atherosclerosis and by contrast, small-diameter vessels were remodelled.

The coronary microvasculature relies on endothelium-dependent nitric oxide signalling for dilation to respond to changes in myocardial demand primarily through metabolic signalling across the vascular endothelium [28]. We previously demonstrated that ILK deletion in vivo induced a lack of NO production by eNOS in response to Ach stimulation, and eNOS need ILK to release NO appropriately [12]. Extensive nitrotyrosine staining in the endothelial ILK deletion model may reflect eNOS uncoupling. Therefore, ILK deletion in coronary microvasculature presumably leads to microvascular endothelial dysfunction. Our results support this hypothesis since the ischaemic events occur early after endothelial ILK deletion, and oxidative markers increase within the first week after deletion. ecILK cKO mice have elevated blood pressure (BP), reflecting the loss of adequate NO supply, since eNOS is essential for regulating blood pressure and hypertension is the most prominent phenotype of eNOS-deficient mice [17, 43]. However, the increased BP was only significant three weeks after endothelial ILK deletion, and although Losartan treatment prevented BP increase, it only partially impeded fibrosis development and did not affect microvascular remodelling. Our results suggest that endothelial ILK deletion markedly targets the small intramural coronary circulation, promoting arteriole remodelling and decreased capillary number. Reduced capillary density also increases intercapillary distance, which may lower the myocardium oxygen supply, making it more susceptible to ischaemia. This parameter has been correlated with a higher risk of heart failure [49]. Thus, the occurrence of mild cardiac hypertrophy after three weeks of ILK deletion may amplify the initial damage caused by microvascular dysfunction [34, 51].

There is increasing evidence that functional and structural alterations in coronary microcirculation (coronary microvascular disfunction, CMD) are responsible for myocardial ischaemia [50]. In patients with chronic obstructive CAD, microvascular dysfunction contributes to myocardial ischaemia in regions supplied by arteries without stenosis and synergistically contributes to myocardial ischaemia in regions with epicardial flow limitation [44]. Moreover, microvascular dysfunction is a recognised complication of acute myocardial ischaemia and is associated with adverse myocardial remodelling, so nowadays is a target of cardioprotection strategies [11, 13, 14, 33, 47]. Finally, microvascular dysfunction is a major cause of myocardial ischaemia in patients with angina without obstructive (CAD) [44]. However, there are no proven therapies available yet, in part due to the lack of animal models. Thus, although ecILK cKO mice may not fully recapitulate human pathophysiology, they still represent a valuable tool for studying coronary microvascular disease.

After the ischaemic injury, the repair programme involves macrophage and myofibroblast activation, which mainly depends on TGFβ1/Smad signalling [10]. The ecILK cKO mice exhibited diffuse interstitial fibrosis. Several possibilities can explain this progressive fibrosis. First, intermittent ischaemic episodes can occur as the heart adapt to ILK loss and, therefore, to decreased endothelial NO production. Although interstitial fibrosis may result from increased blood pressure, the antihypertensive Losartan only partially prevented fibrosis development. As an inhibitor of the angiotensin II receptor, Losartan may counteract the effect of the lack of NO, preventing the development of reactive interstitial fibrosis [21]. However, Losartan treatment did not reduce microvascular remodelling, indicating that ILK specifically protects the microvasculature. Second, microvascular disease decreases coronary reserve, compromising cardiomyocyte survival, and may account for the myocardial scarring found in our model [53]. As angiogenesis in vivo is initiated in micro-vessels, loss of microvascular endothelial cell function will impair and delay the growth of blood vessels necessary for the reparative process after a myocardial injury [8]. Lastly, small intramural arteriole remodelling can lead to further ischaemia in areas distal from the initial ischaemic injury site, causing myofibroblasts to remain in a persistent profibrogenic state instead of returning to a quiescent phenotype. In our model, MMP-9, -2 and -13, fibrillar collagen (I and III), TGF-β1 and Smad2/3 expression increased, indicating an active remodelling process. NO regulates TGF-β1 effects in endothelial cells [41, 45]; thus, ILK may affect ECM turnover via decreased NO signalling leading to fibrosis [4, 36]. Alternatively, since ILK acts as a mechanotransducer, altered ECM may affect fibroblasts synthetic profile and differentiation to myofibroblasts [27].

Endothelial-to-mesenchymal transition (endMT) is emerging as a potential mechanism responsible for the increase in the fibroblast population and may increase fibrosis [55]. In our study, the most relevant pathways that predicted by the Ingenuity pathway analysis, Pulmonary fibrosis, Transforming growth factor signalling and Hippo pathway, were all of them related to EndMT and ILK pathways.

EndMT is characterised by a phenotypic change of quiescent endothelial cells that adopt mesenchymal cells' shape and behaviour [1]. Here, we demonstrate that endothelial ILK deletion induces the loss of endothelial cell markers acquiring a mesenchymal cell phenotype. Surprisingly, many cells undergoing endMT expressed endothelial markers and mesenchymal markers simultaneously, suggesting a partial transition. Recently, a study in critical limb ischaemia reported partial endMT as a mechanism of microvascular luminal narrowing [3]. In addition, several reports describe endMT as a critical mechanism in the occlusive vascular remodelling observed in pulmonary artery hypertension [6]. Indeed, many arterioles in ecILK cKO hearts presented significant luminal stenosis, and some were entirely occluded. Micro-vessel obstruction may result from thrombotic material released due to haemodynamic disturbances. In addition, soluble substances released into the coronary circulation (i.e. endothelin-1) may facilitate vasoconstriction. Coronary microvascular obstruction and dysfunction result in patchy micro-infarctions accompanied by an inflammatory response, as was detected by TTC staining and immunohistochemistry in the heart sections of ecILK cKO mice. Both may contribute to progressive myocardial contractile dysfunction [22].

Transforming growth factor and haemodynamic perturbations are the two main triggers of EndMT [47, 55]. These conditions are present in ecILK cKO mice and could contribute to the observed structural and functional effects. Microvascular stenosis induced-low shear could contribute to micro-vessel inward remodelling affecting smooth muscle cells. Indeed, in chronic hypoxia in pulmonary hypertension, decreased ILK expression is involved in regulating the vascular SMC phenotype [16]. It is possible that endothelial ILK deletion also regulates SMC phenotype or increases vasoconstrictor responses. Additionally, reduced endothelial NO synthesis by ILK-deleted endothelial cells may contribute to endMT. As mentioned before, NO regulates TGF-β1 effects in endothelial cells [41, 45] and we have recently demonstrated that ILK deletion in human aortic valve endothelial cells leads to endMT in a TGF-β and NO-dependent manner [39]. Thus, it is possible that TGF/NO axis also plays a role in the effects observed.

The endothelial phenotype shift and the associated luminal stenosis constitute a previously unrecognised arteriolar remodelling process that could exacerbate impaired myocardium perfusion, contributing to myocardial ischaemia and adverse outcomes [19]. Our data provide a background of the cardioprotective effect of endothelial ILK in cardiac ischaemia and, with limitations, provide a valuable tool to study microvascular disease. Understanding in greater depth, the mechanisms that govern ILK expression in the coronary endothelium can improve the knowledge of the pathogenesis of microvascular dysfunction and prevent progressive loss of viable myocardium. More importantly, finding a reliable marker of decreased ILK expression on coronary endothelium would be an interesting first step to preventing its decline.

### Supplementary Information

Below is the link to the electronic supplementary material.

Supplementary file1 (PPTX 22147 KB)

Supplementary file2 (PPTX 1702 KB)

Supplementary file3 (DOCX 37 KB)

## Data Availability

Data supporting this study will be available to qualified researchers upon reasonable request.
