# Supplementary material for: Endothelial ILK induces cardioprotection by preventing coronary microvascular dysfunction and endothelial-to-mesenchymal transition
Source: Basic Res Cardiol. 2023 Jul 14;118(1):28. doi: 10.1007/s00395-023-00997-0 (PMC10348984; doi:10.1007/s00395-023-00997-0)
Supplement: Supplementary file 1 — Supplementary file1 (PPTX 22147 KB) [file 395_2023_997_MOESM1_ESM.pptx]

## Slide 1
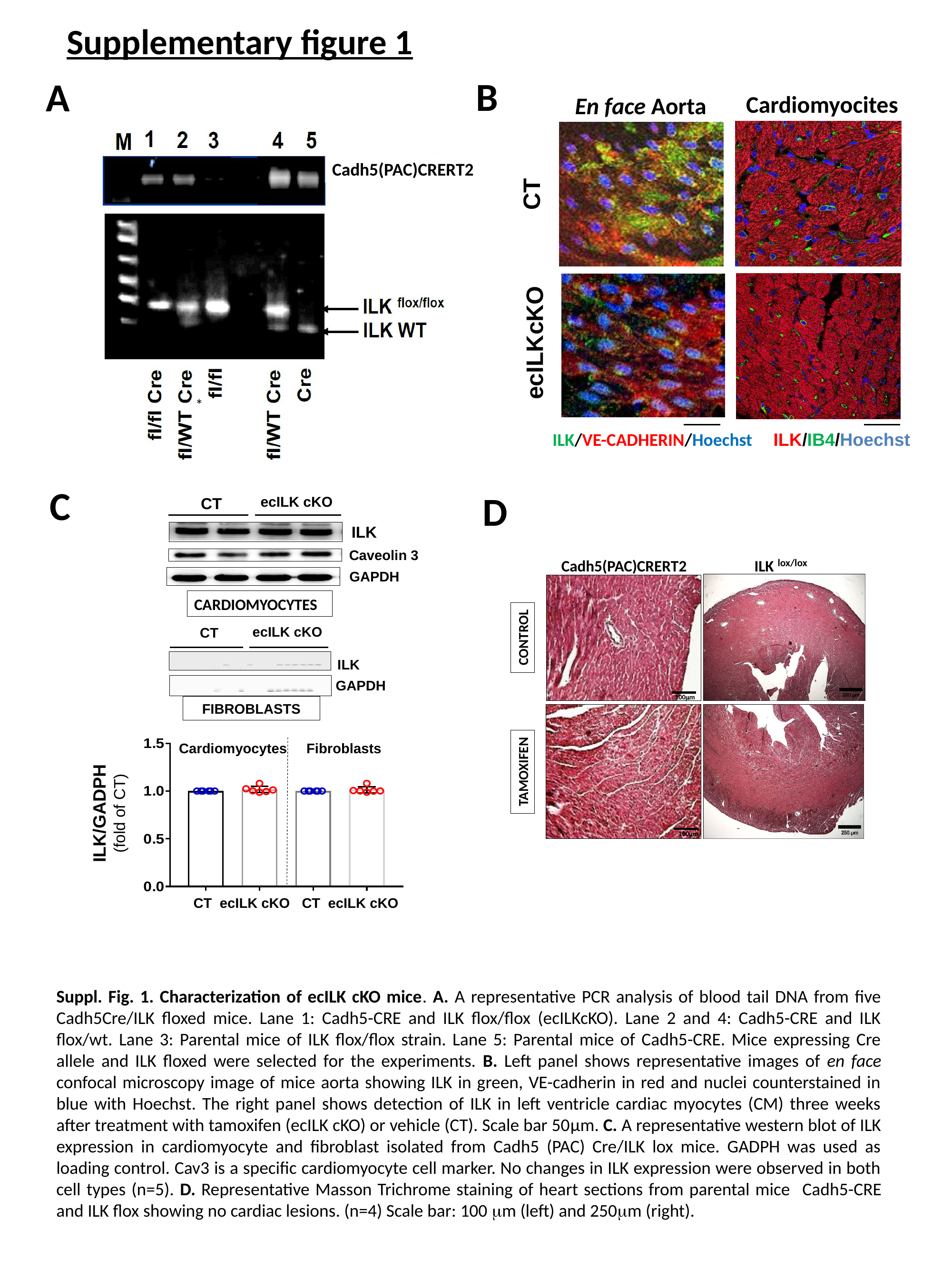

Supplementary figure 1
B
A
Cardiomyocites
En face Aorta
CT
ecILKcKO
ILK/VE-CADHERIN/Hoechst
ILK/IB4/Hoechst
Cadh5(PAC)CRERT2
*
C
D
ecILK cKO
CT
ILK
Caveolin 3
GAPDH
CARDIOMYOCYTES
Cadh5(PAC)CRERT2 ILK lox/lox
CONTROL
100mm
TAMOXIFEN
100mm
ecILK cKO
CT
ILK
GAPDH
FIBROBLASTS
Cardiomyocytes Fibroblasts
ILK/GADPH
(fold of CT)
CT ecILK cKO
CT ecILK cKO
Suppl. Fig. 1. Characterization of ecILK cKO mice. A. A representative PCR analysis of blood tail DNA from five Cadh5Cre/ILK floxed mice. Lane 1: Cadh5-CRE and ILK flox/flox (ecILKcKO). Lane 2 and 4: Cadh5-CRE and ILK flox/wt. Lane 3: Parental mice of ILK flox/flox strain. Lane 5: Parental mice of Cadh5-CRE. Mice expressing Cre allele and ILK floxed were selected for the experiments. B. Left panel shows representative images of en face confocal microscopy image of mice aorta showing ILK in green, VE-cadherin in red and nuclei counterstained in blue with Hoechst. The right panel shows detection of ILK in left ventricle cardiac myocytes (CM) three weeks after treatment with tamoxifen (ecILK cKO) or vehicle (CT). Scale bar 50μm. C. A representative western blot of ILK expression in cardiomyocyte and fibroblast isolated from Cadh5 (PAC) Cre/ILK lox mice. GADPH was used as loading control. Cav3 is a specific cardiomyocyte cell marker. No changes in ILK expression were observed in both cell types (n=5). D. Representative Masson Trichrome staining of heart sections from parental mice Cadh5-CRE and ILK flox showing no cardiac lesions. (n=4) Scale bar: 100 mm (left) and 250mm (right).

## Slide 2
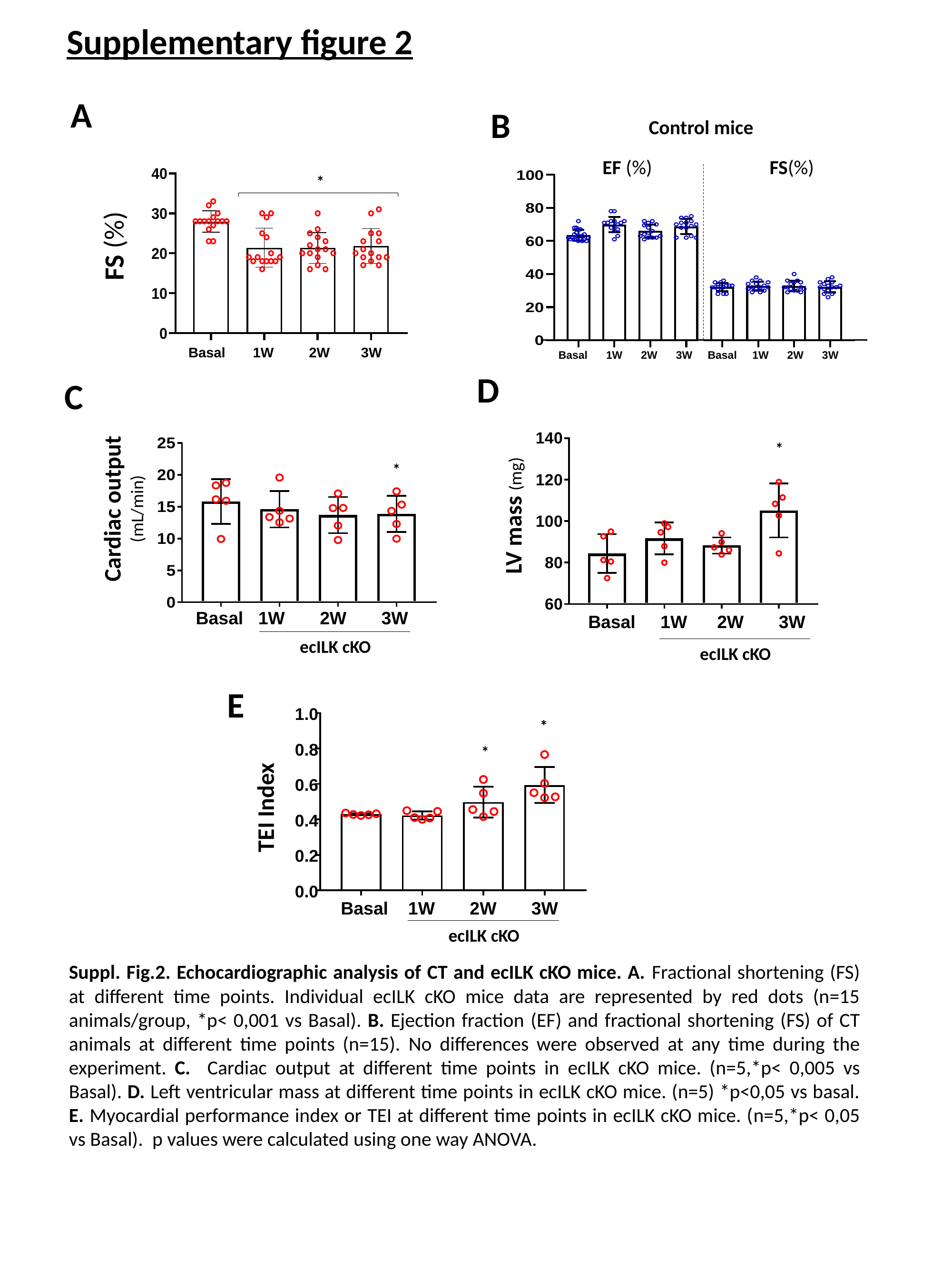

Supplementary figure 2
A
B
Control mice
EF (%) FS(%)
Basal 1W 2W 3W
*
FS (%)
Basal 1W 2W 3W
Basal 1W 2W 3W
D
C
*
LV mass (mg)
 Basal 1W 2W 3W
*
Cardiac output
(mL/min)
Basal 1W 2W 3W
ecILK cKO
ecILK cKO
E
1.0
*
0.8
TEI Index
Basal 1W 2W 3W
ecILK cKO
0.6
0.4
0.2
0.0
*
Suppl. Fig.2. Echocardiographic analysis of CT and ecILK cKO mice. A. Fractional shortening (FS) at different time points. Individual ecILK cKO mice data are represented by red dots (n=15 animals/group, *p< 0,001 vs Basal). B. Ejection fraction (EF) and fractional shortening (FS) of CT animals at different time points (n=15). No differences were observed at any time during the experiment. C. Cardiac output at different time points in ecILK cKO mice. (n=5,*p< 0,005 vs Basal). D. Left ventricular mass at different time points in ecILK cKO mice. (n=5) *p<0,05 vs basal. E. Myocardial performance index or TEI at different time points in ecILK cKO mice. (n=5,*p< 0,05 vs Basal). p values were calculated using one way ANOVA.

## Slide 3
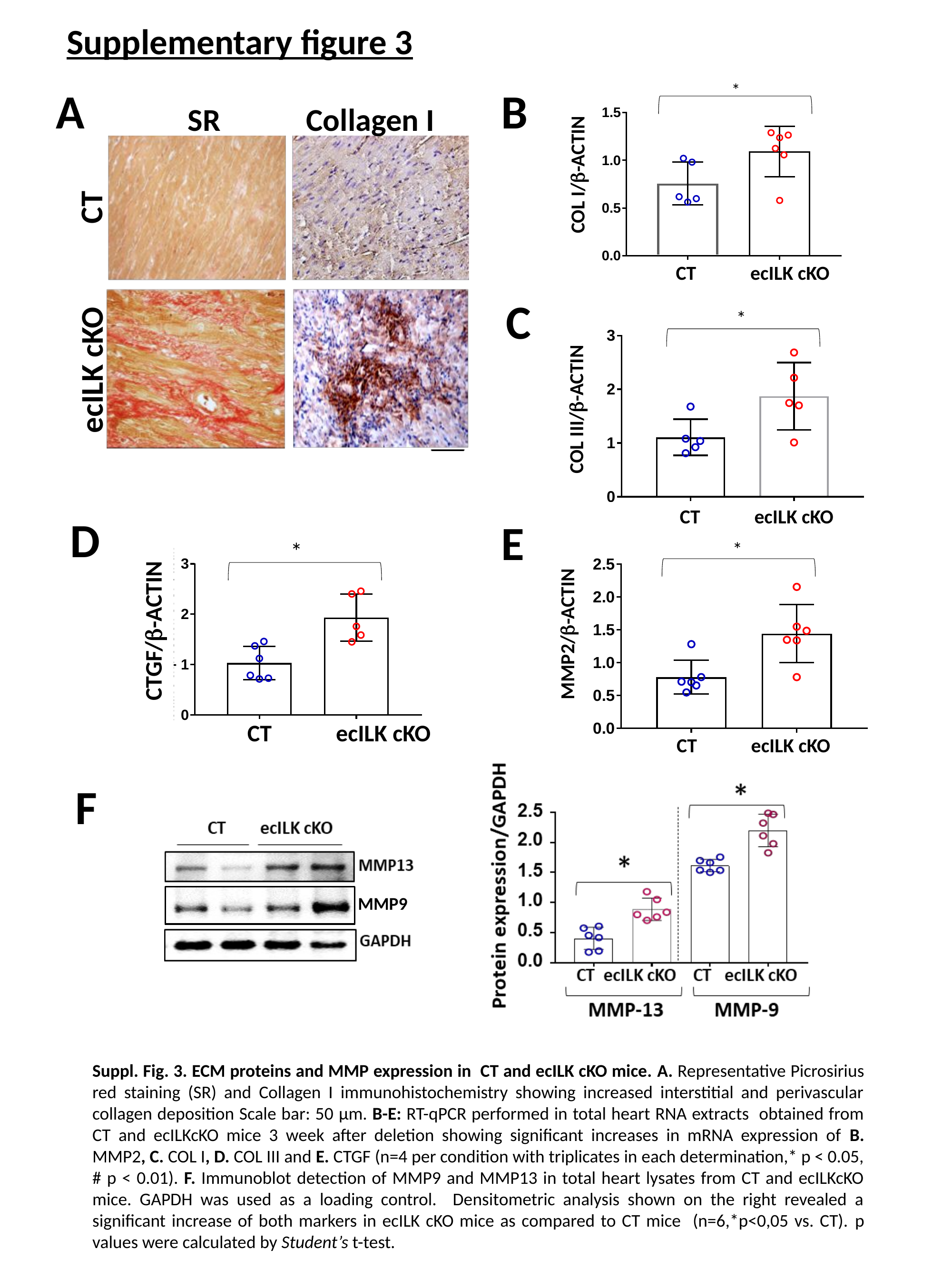

Supplementary figure 3
*
A
B
COL I/b-ACTIN
CT ecILK cKO
SR Collagen I
CT
ecILK cKO
C
*
COL III/b-ACTIN
CT ecILK cKO
D
E
*
CTGF/b-ACTIN
CT ecILK cKO
*
MMP2/b-ACTIN
CT ecILK cKO
F
MMP9
Suppl. Fig. 3. ECM proteins and MMP expression in CT and ecILK cKO mice. A. Representative Picrosirius red staining (SR) and Collagen I immunohistochemistry showing increased interstitial and perivascular collagen deposition Scale bar: 50 µm. B-E: RT-qPCR performed in total heart RNA extracts obtained from CT and ecILKcKO mice 3 week after deletion showing significant increases in mRNA expression of B. MMP2, C. COL I, D. COL III and E. CTGF (n=4 per condition with triplicates in each determination,* p < 0.05, # p < 0.01). F. Immunoblot detection of MMP9 and MMP13 in total heart lysates from CT and ecILKcKO mice. GAPDH was used as a loading control. Densitometric analysis shown on the right revealed a significant increase of both markers in ecILK cKO mice as compared to CT mice (n=6,*p<0,05 vs. CT). p values were calculated by Student’s t-test.

## Slide 4
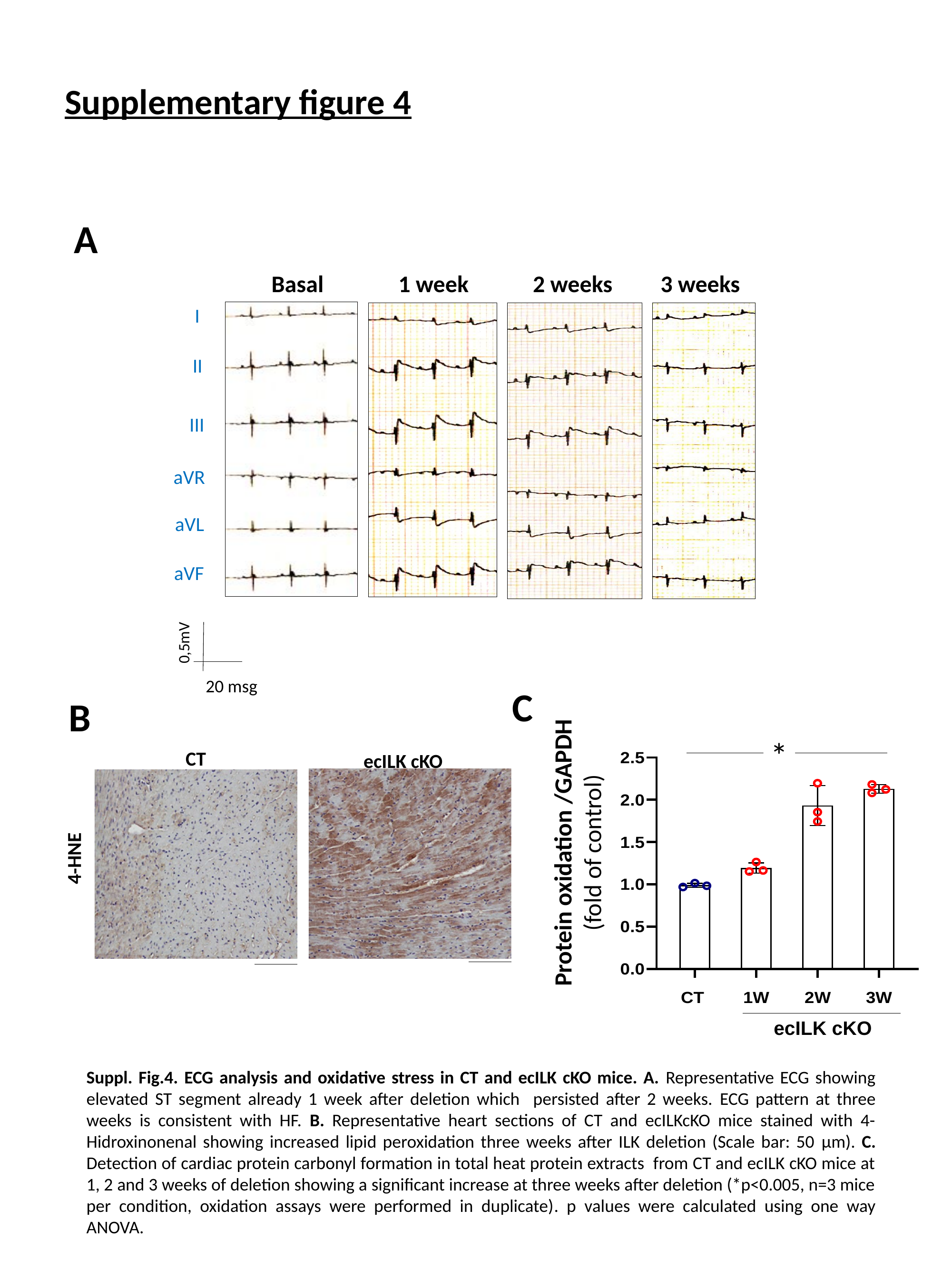

Supplementary figure 4
A
Basal 1 week 2 weeks 3 weeks
I
II
III
aVR
aVL
aVF
0,5mV
20 msg
C
B
*
Protein oxidation /GAPDH
(fold of control)
ecILK cKO
CT
ecILK cKO
4-HNE
Suppl. Fig.4. ECG analysis and oxidative stress in CT and ecILK cKO mice. A. Representative ECG showing elevated ST segment already 1 week after deletion which persisted after 2 weeks. ECG pattern at three weeks is consistent with HF. B. Representative heart sections of CT and ecILKcKO mice stained with 4-Hidroxinonenal showing increased lipid peroxidation three weeks after ILK deletion (Scale bar: 50 μm). C. Detection of cardiac protein carbonyl formation in total heat protein extracts from CT and ecILK cKO mice at 1, 2 and 3 weeks of deletion showing a significant increase at three weeks after deletion (*p<0.005, n=3 mice per condition, oxidation assays were performed in duplicate). p values were calculated using one way ANOVA.

## Slide 5
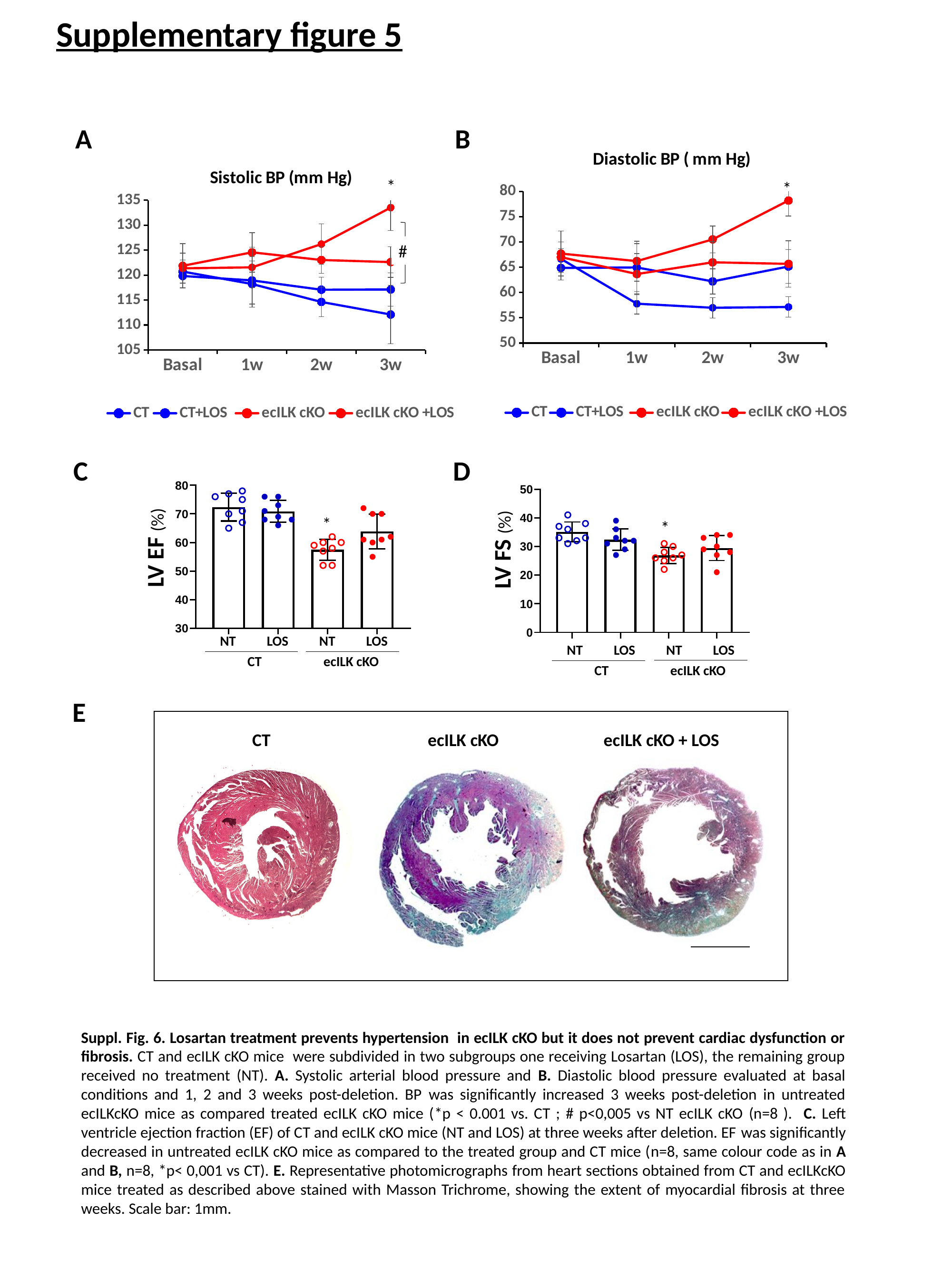

Supplementary figure 5
A
B
### Chart: Diastolic BP ( mm Hg)
| Category | CT | CT+LOS | ecILK cKO | ecILK cKO +LOS |
|---|---|---|---|---|
| Basal | 64.85999999999999 | 66.65 | 67.7 | 66.99 |
| 1w | 64.955 | 57.787499999999994 | 66.204 | 63.645999999999994 |
| 2w | 62.2 | 56.98 | 70.5 | 65.97142857142856 |
| 3w | 65.14 | 57.137499999999996 | 78.18666666 | 65.66054421428571 |
### Chart: Sistolic BP (mm Hg)
| Category | CT | CT+LOS | ecILK cKO | ecILK cKO +LOS |
|---|---|---|---|---|
| Basal | 119.816666666667 | 120.7 | 121.35000000000001 | 121.84999999999998 |
| 1w | 118.93 | 118.20499999999998 | 121.545 | 124.55 |
| 2w | 117.06666666666666 | 114.62 | 126.2 | 123.01666666666667 |
| 3w | 117.11666666666667 | 112.09999999999998 | 133.49999999999997 | 122.59603166666666 |*
#
*
C
D
*
LV EF (%)
NT LOS NT LOS
CT ecILK cKO
*
LV FS (%)
NT LOS NT LOS
CT ecILK cKO
E
CT ecILK cKO ecILK cKO + LOS
Suppl. Fig. 6. Losartan treatment prevents hypertension in ecILK cKO but it does not prevent cardiac dysfunction or fibrosis. CT and ecILK cKO mice were subdivided in two subgroups one receiving Losartan (LOS), the remaining group received no treatment (NT). A. Systolic arterial blood pressure and B. Diastolic blood pressure evaluated at basal conditions and 1, 2 and 3 weeks post-deletion. BP was significantly increased 3 weeks post-deletion in untreated ecILKcKO mice as compared treated ecILK cKO mice (*p < 0.001 vs. CT ; # p<0,005 vs NT ecILK cKO (n=8 ). C. Left ventricle ejection fraction (EF) of CT and ecILK cKO mice (NT and LOS) at three weeks after deletion. EF was significantly decreased in untreated ecILK cKO mice as compared to the treated group and CT mice (n=8, same colour code as in A and B, n=8, *p< 0,001 vs CT). E. Representative photomicrographs from heart sections obtained from CT and ecILKcKO mice treated as described above stained with Masson Trichrome, showing the extent of myocardial fibrosis at three weeks. Scale bar: 1mm.

## Slide 6
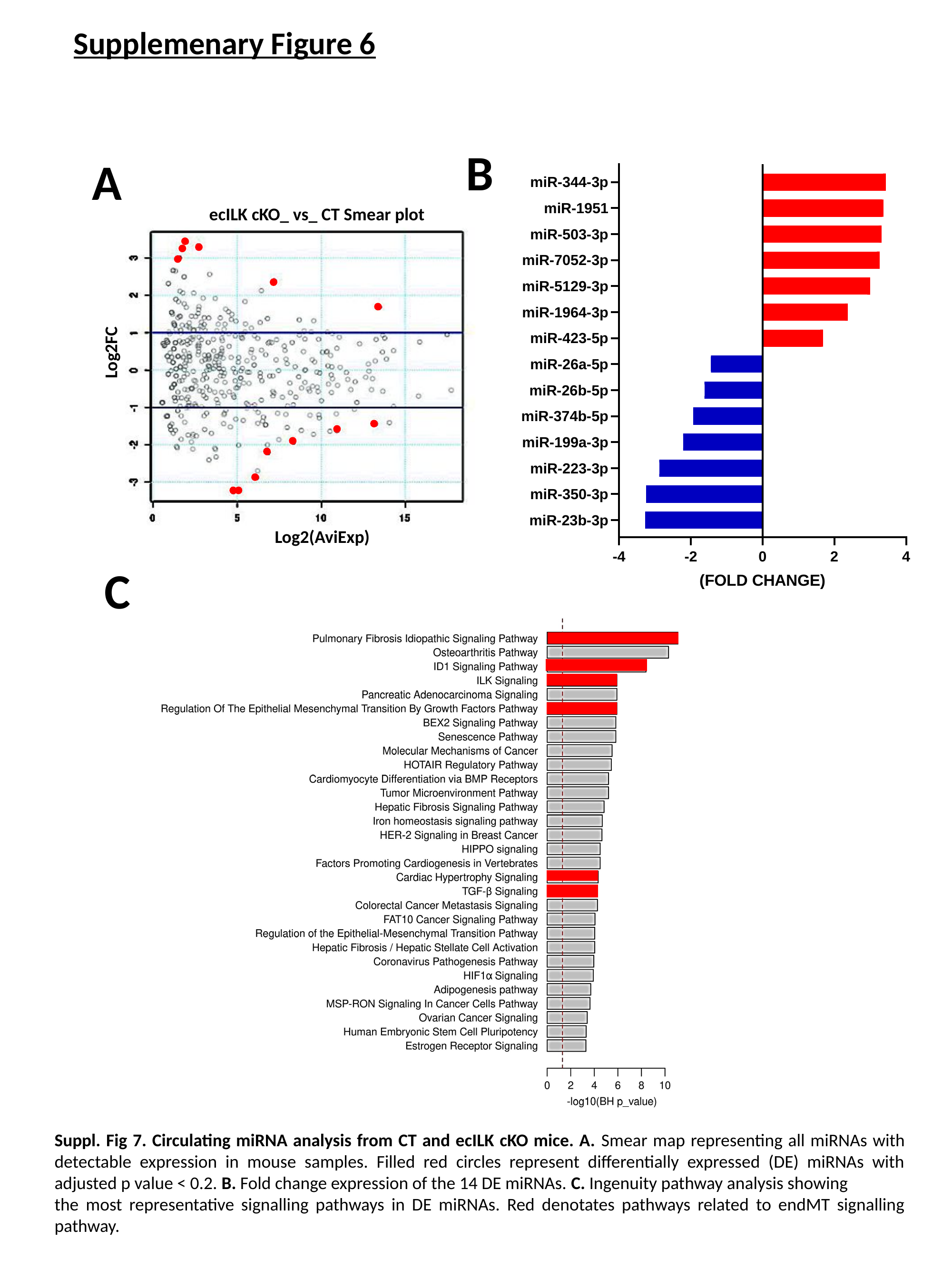

Supplemenary Figure 6
B
A
ecILK cKO_ vs_ CT Smear plot
Log2FC
Log2(AviExp)
C
Suppl. Fig 7. Circulating miRNA analysis from CT and ecILK cKO mice. A. Smear map representing all miRNAs with detectable expression in mouse samples. Filled red circles represent differentially expressed (DE) miRNAs with adjusted p value < 0.2. B. Fold change expression of the 14 DE miRNAs. C. Ingenuity pathway analysis showing
the most representative signalling pathways in DE miRNAs. Red denotates pathways related to endMT signalling pathway.
