# Supplementary material for: Endothelial ILK induces cardioprotection by preventing coronary microvascular dysfunction and endothelial-to-mesenchymal transition
Source: Basic Res Cardiol. 2023 Jul 14;118(1):28. doi: 10.1007/s00395-023-00997-0 (PMC10348984; doi:10.1007/s00395-023-00997-0)
Supplement: Supplementary file 2 — Supplementary file2 (PPTX 1702 KB) [file 395_2023_997_MOESM2_ESM.pptx]

## Slide 1
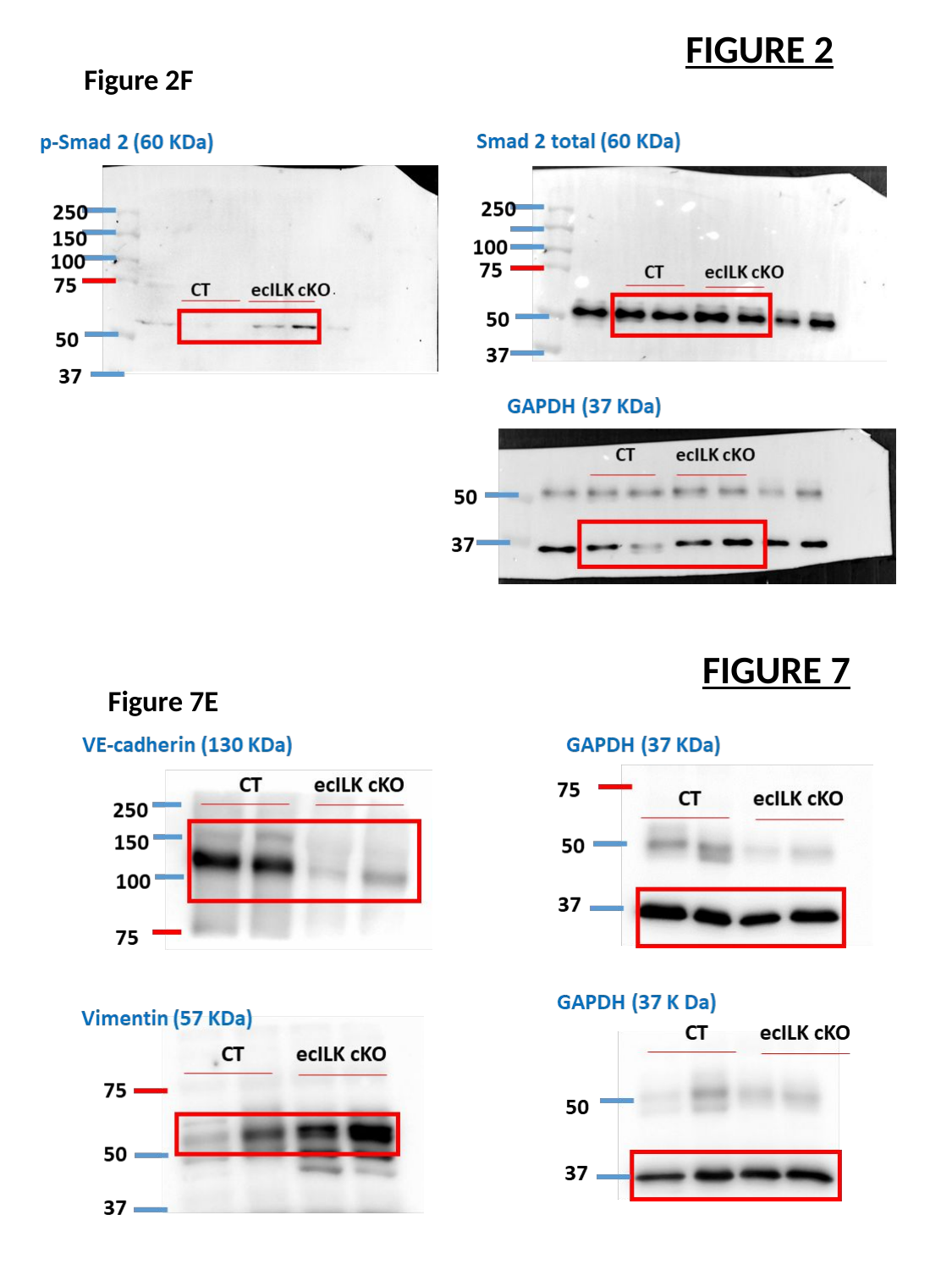

FIGURE 2
Figure 2F
FIGURE 7
Figure 7E

## Slide 2
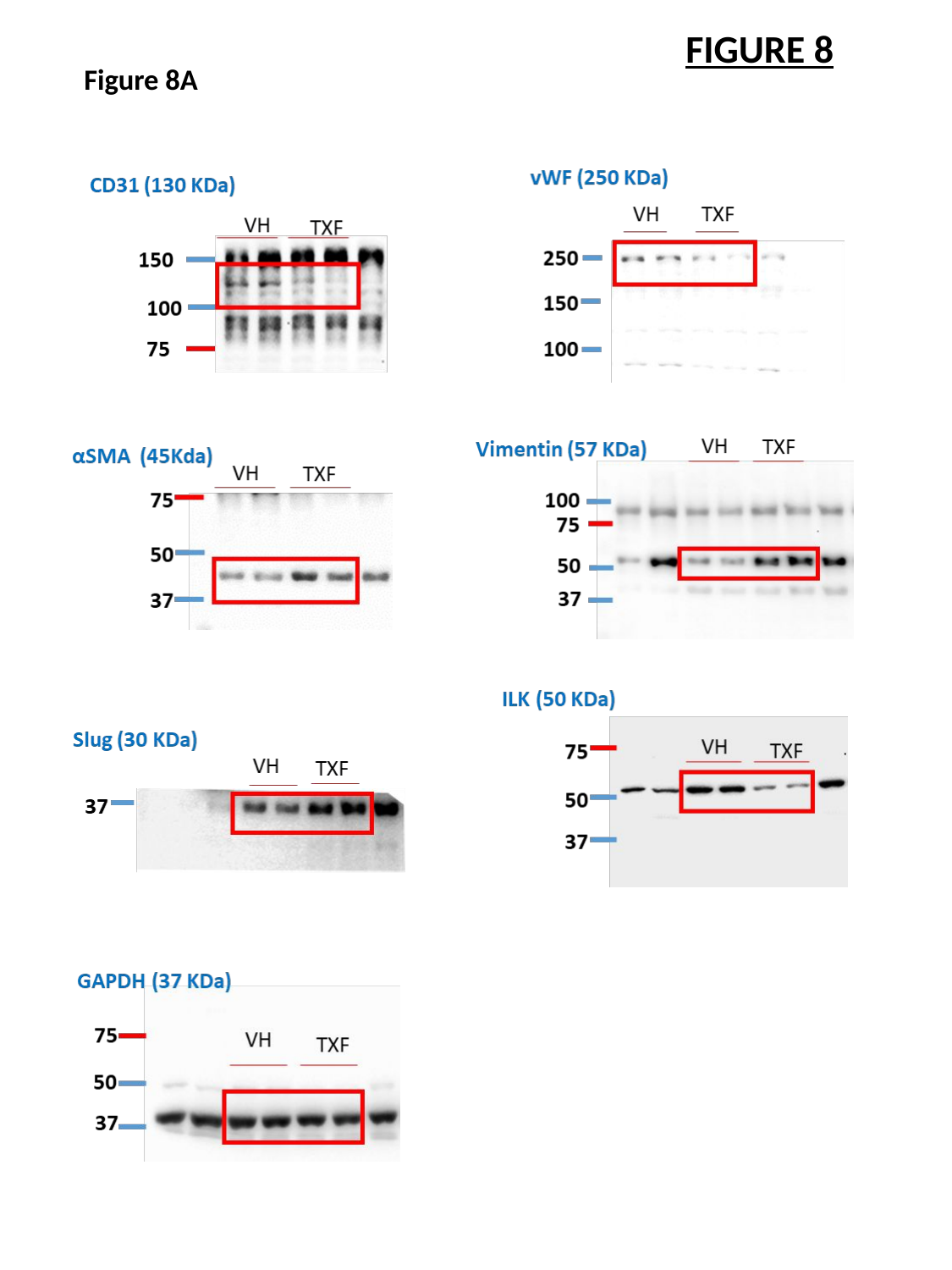

FIGURE 8
Figure 8A

## Slide 3
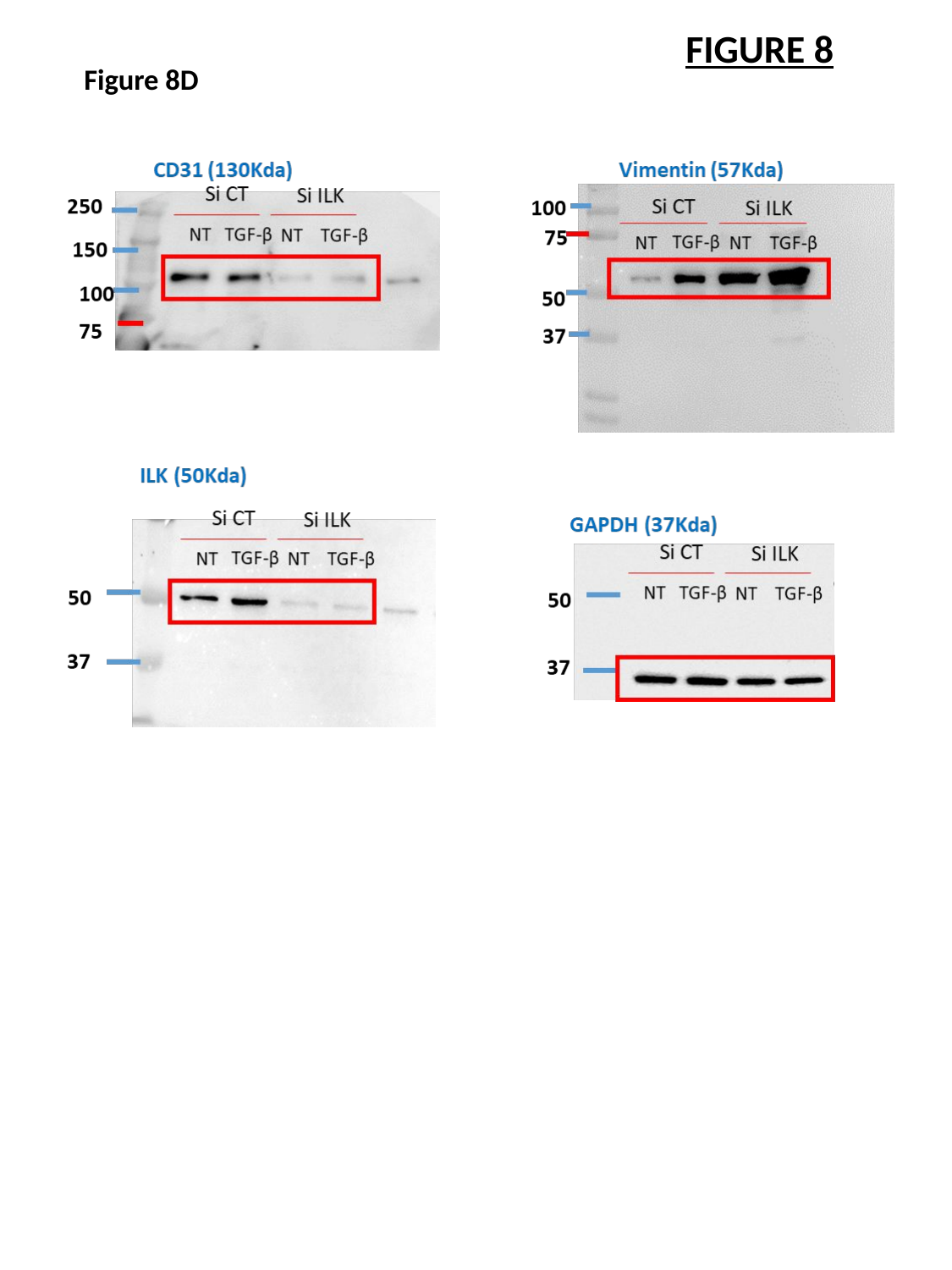

FIGURE 8
Figure 8D

## Slide 4
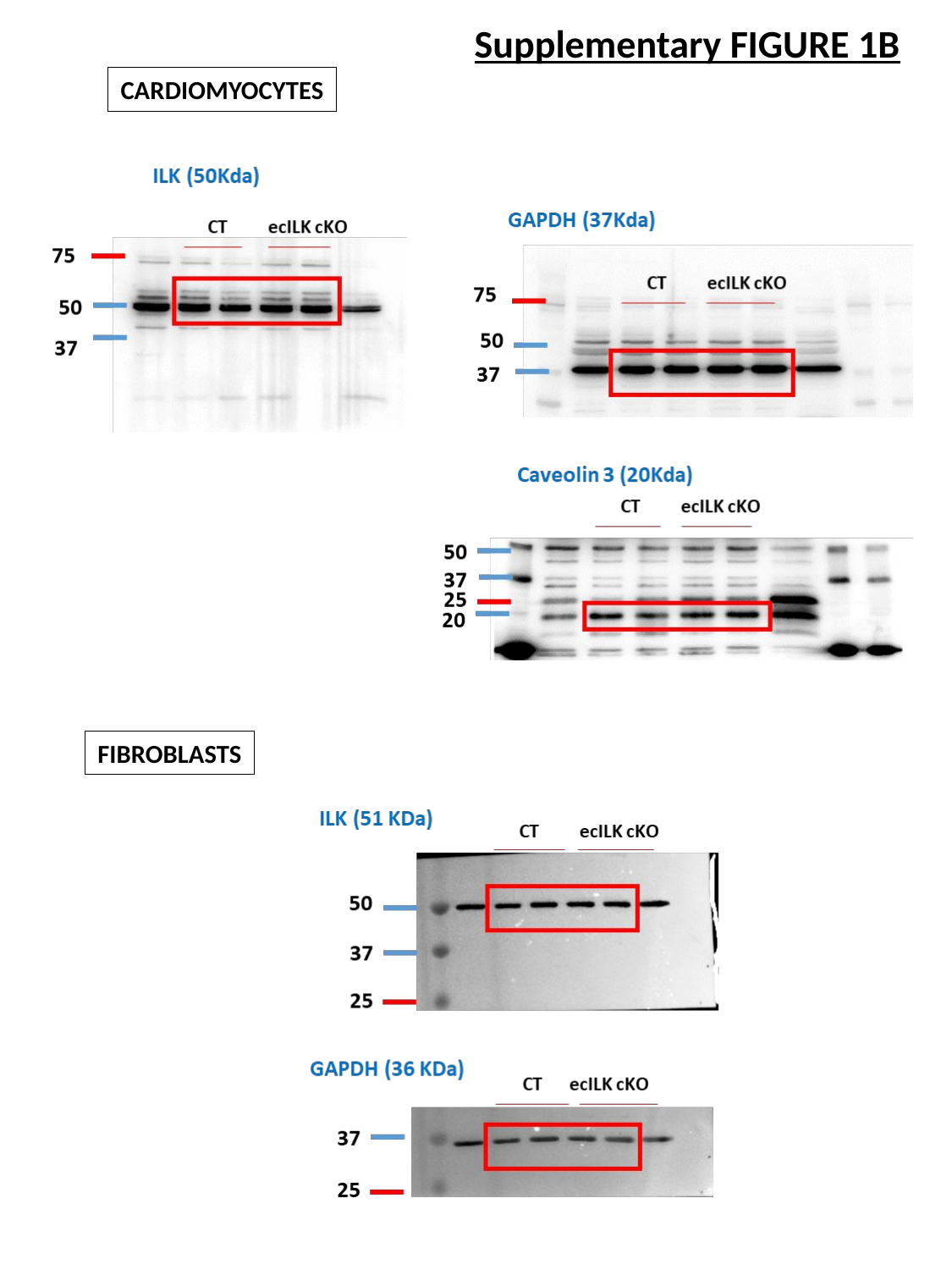

Supplementary FIGURE 1B
CARDIOMYOCYTES
FIBROBLASTS

## Slide 5
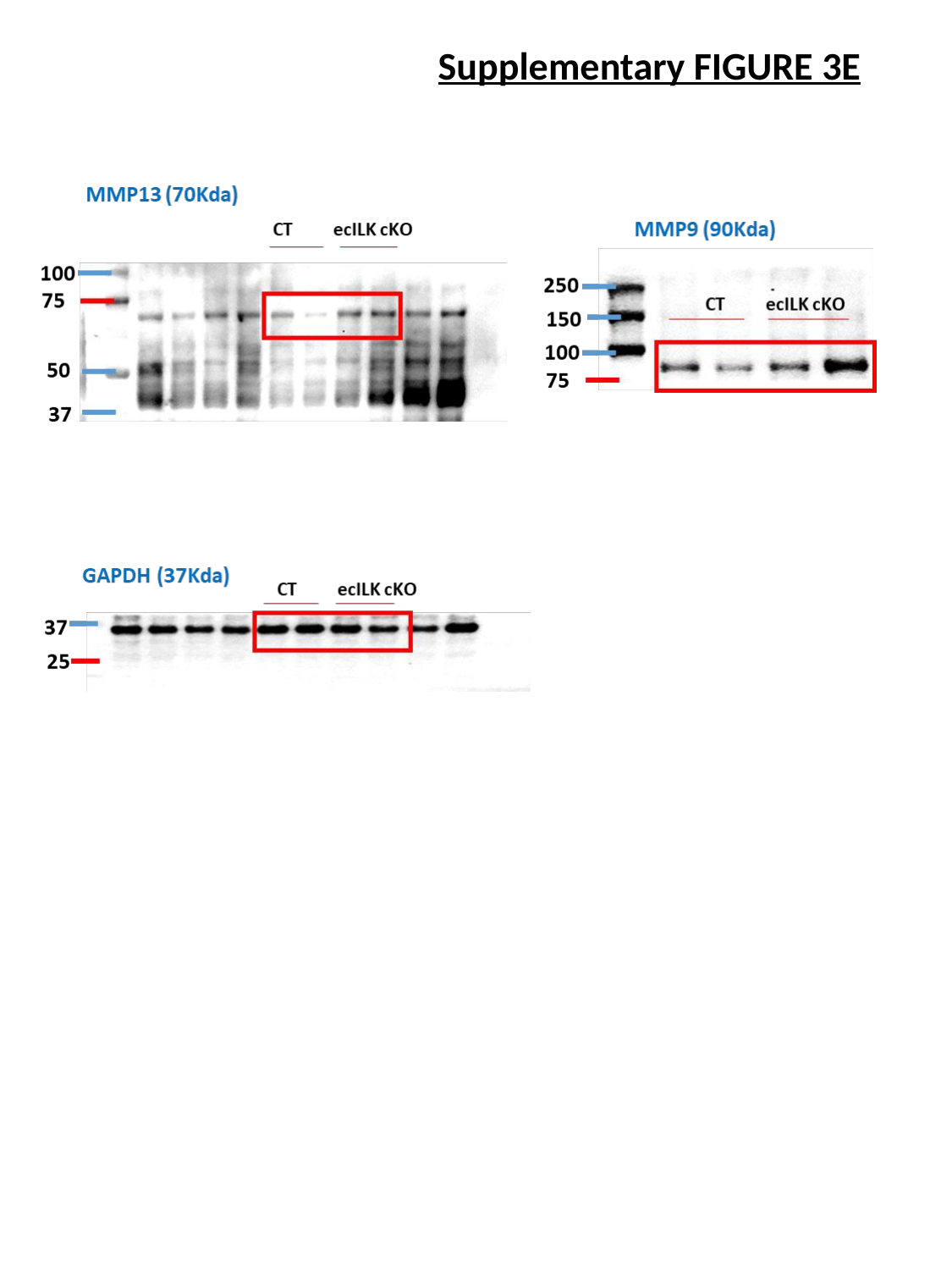

Supplementary FIGURE 3E
