# Supplementary material for: Endothelial ILK induces cardioprotection by preventing coronary microvascular dysfunction and endothelial-to-mesenchymal transition
Source: Basic Res Cardiol. 2023 Jul 14;118(1):28. doi: 10.1007/s00395-023-00997-0 (PMC10348984; doi:10.1007/s00395-023-00997-0)
Supplement: Supplementary file 3 — Supplementary file3 (DOCX 37 KB) [file 395_2023_997_MOESM3_ESM.docx]

**BASIC RESEARCH IN CARDIOLOGY** **R1**

**Endothelial ILK induces cardioprotection by preventing coronary microvascular dysfunction and endothelial-to-mesenchymal transition.**

Reventun P^1,2*^, Sanchez:Esteban S^1*^, Cook A^1^, Delgado Marin, M^1^, Roza C^1^,Jorquera-Ortega S^1^, Hernandez I^3^, Tesoro L^3^, Botana L^3^, Zamorano JL^4,5^, Zaragoza C^3,5^, Saura M^1,5$^

**Affiliations:**

1 Universidad Alcalá, Facultad Medicina, Depto. Biología Sistemas (UD Fisiología). IRYCIS

2 Johns Hopkins University. School of Medicine. Department of Medicine. Cardiology Division (present address)

3Unidad Mixta de Investigación Cardiovascular, Universidad Francisco de Vitoria, Pozuelo de Alarcón, Madrid, Spain

4 Servicio Cardiología. Hospital Universitario Ramón y Cajal, Madrid, Spain

5Centro de Investigación Biomédica en Red en Enfermedades Cardiovasculares (CIBERCV), Spain

***** These authors contributed equally to this work.

^$^ **Corresponding author:** [marta.saura@uah.es](mailto:marta.saura@uah.es)

Universidad Alcalá, Fac. Medicina, Dept. Biología Sistemas (Fisiología). Mod 2 planta 0. Ctra Madrid.: Barcelona km 33,500. Alcalá de Henares Madrid

**Supplemental Material and Methods**

**Reagents and Antibodies**

General cell culture supplies were purchased from Lonza (Basel, Switzerland); fetal bovin serum was from Gibco (Waltham, Ma, USA). Matrigel and Dispase from BD Biosciences (San José, Ca, USA). Cell culture-grade gelatin, hematoxylin-eosin, fluorescein isothiocyanate (FITC)-dextran, KAPA HotStart Mouse Genotyping Kit, OxyBlot Protein Oxidation Detection Kit, Direct Red 80, Picric acid Saturated aqueous solution (1.3% in water), Thioflavin S, 2,3,5-Triphenyltetrazolium chloride (TTC), and 4-hydroxy-tamoxifen for cell treatment were from Sigma Aldrich (San Luis, MO, USA). Optimum cutting temperature (OCT) was from Sakura Finetek (Torrance, CA, USA). Tamoxifen for animal treatment was from Tocris Bioscience (Bristol, UK). Carazzi hematoxylin and DPX were from Casa Alvarez (Madrid, Spain). Masson’s trichrome staining kit EMD Millipore Corporation (Burlintong, Ma, USA). Mouse and Rabbit Specific HRP/DAB (ABC) Detection IHC kit was from Abcam. SuperSignal detection system Pierce™ ECL Western Blotting Substrate (Waltham, MA, USA). TRIzol reagent, RT-PCR kit, and FITC–conjugated wheat germ agglutinin (WGA) from Invitrogen Corporation (Carlsbad, CA, USA). Complet mini and phosphostop were from Roche (Switzerland). A detail listing of the antibodies used through the study is provided bellow.

| Antibody | Supplier | Reference | Dilution |  |
| --- | --- | --- | --- | --- |
| Anti:Ve:cadherin | Abcam | ab7047 | *En face*: 1:50 | |
|  | Santa cruz | sc:9989 | WB: 1:250  IF: 1:50 | |
| Anti:ILK | Cell Signaling | 3856S | *En face*: 1:100 | |
|  | RD Systems | MAB374 | WB: 1:1000 | |
|  | Bioss | BS:0317R | IF, Flow cytometry: 1:50 | |
| Anti:CD31 | Abcam | ab32457 | WB: 1:250  Flow cytometry: 1:50 | |
|  | RD Systems | AF3628 | IF: 1:75 | |
| Anti: α:smooth muscle actin | \| Thermo Scientific \|  \| \| --- \| --- \| | MA1:06110 | IF, Flow cytometry:1:100  WB:1:1000 | |
| Anti:GAPDH | Millipore | MAB374 | WB: 1:1000 | |
| Alexa Fluor 488–conjugated wheat germ agglutinin | Life Technologies | W11261 | IF: 1:100 | |
| anti:GSl I:isoleoctin B4 (IB4) | Vector Laboratories | FL:1201 | IF: 1:50 | |
| anti:GSl I:isoleoctin B4 (IB4) | Vector Laboratories | DL-1207 | IF: 1:50 | |
| Anti:Collagen I | Abcam | ab34710 | IHC:1:50 | |
| Anti:TGFβ | Santa Cruz | sc:130348 | IHC:1:50 | |
| Anti:Smad2/3 | BD Biosciences | 610842 | IF: 1:50  WB: 1:500 | |
| Anti:phospho Smad2 | Cell Signaling | 18338 | WB: 1:500 | |
| Anti:MMP 9 | Santa Cruz | sc:6840 | WB: 1:500 | |
| Anti:MMP 13 | Santa Cruz | sc:515284 | WB: 1:500 | |
| Anti:Caveolin 3 | Abcam | ab289544 | WB: 1:500 | |
| 4 Hidroxinonenal | Abcam | ab46545 | IHC: 1:50 | |
| Anti:Slug | Cell Signaling | 9585S | WB: 1:500 | |
| Anti:vWF | Santa Cruz | sc:365712 | WB:1:500 | |
| Anti:Fibronectin | Merck | AB2033 | IF: 1:50 | |
| Anti:Nitrotyrosine | Millipore | 06:284 | IF: 1:50 | |
| Anti:Vimentin | Abcam | ab92547 | WB: 1:1000  IF: 1:100 | |
| HRP:conjugated anti:Mouse secondary antibody | Invitrogen | A16072 | WB: 1:20000 | |
| HRP:conjugated anti:Rabbit secondary antibody | Invitrogen | 31466 | WB: 1:50000 | |
| Alexa fluor 488 conjugated Goat anti:Rabbit secondary antibody | Abcam | ab150081 | IF,Flow Cytometry:1:500 | |
| Alexa fluor 647 conjugated Goat anti:mouse secondary antibody | Abcam | ab150115 | IF, Flow Cytometry:1:500 | |
| Alexa fluor 647 conjugated Goat anti:Rabbit secondary antibody | Abcam | ab150083 | IF, Flow Cytometry:1:500 | |
| Alexa fluor 488 conjugated Donkey anti:Goat secondary antibody | Abcam | ab150129 | IF: 1:500 | |
| Alexa fluor 647 conjugated Donkey anti: Rabbit secondary antibody | Abcam | ab150075 | IF: 1:500 | |

**Mouse genotyping**

Endothelial ILK conditional knockout mice (ecILK cKO) were genotyped by tail DNA analysis using KAPA HotStart Mouse Genotyping Kit (Sigma) and following manufacture instructions. DNA amplification was performed by Polymerase chain reaction (PCR) using the following conditions: Initial denaturation at 95ºC, 5 min following 35 cycles of denaturalization (95ºC, 45 sec), primer annealing (57ºC, 30 sec), and extension (72ºC, 45 sec), and a final extension of 10 min at 72ºC. Specific primer were **VE:cad:Cre forward** 5':CTG GGA TGC TGA GGC ATC AG :3', **VE:cad:Cre reverse** 5': TTG CGA ACC TCA TCA CTC GTT :3' yielding a 760:bp fragment, and **ILK forward** 5’: GTC TTG CAA ACC CGT CTC TGC G :3', **ILK reverse** 5':CAG AGG TGT CAG TGC TGG GAT G :3' yielding amplimers of 370:bp for the ILK floxed mice (ILK^fl/fl^), amplimers of 395:bp for the ILK WT mice (ILK^wt/wt^) and amplimers of 370:bp and 395:bp for the heterocygous phenotype (ILK^fl/wt^).

**Cell transfection**

Cells were transfected with 25 nM non:targeting siRNA(si:CT) or ILK:targeting siRNA (si:ILK) (Santa Cruz Biotech, Santa Cruz, CA, USA)45 using Lipofectamine 2000 transfection reagent and Opti:MEM (Gibco, Waltham, Ma, USA) for 6 hour. Following 24 hours transfection, cells were treated with 2ng/ml TFG:β for 3 days at 37 °C, 5% CO2.

***En face* Aorta**

For the Immunohistochemical measurement of endothelial ILK expression *ex vivo* mouse aortas were extracted from 12 week:old ecILK cKO and CT cKO. Aortic rings were cut into 4 mm-thick segments on ice and fixed in 4% paraformaldehyde as describe elsewhere [1]. Tissue was permeabilizated with Triton 0.3%, blocked with BSA 2% and incubated with antibodies to ILK (Cell Signalling) and the endothelial marker Ve:cadherin (Abcam) overnight at 4ºC. The rings were then washed three times, incubated with secondary antibodies (Alexa-488 and Alexa-647) and stained with Hoechst for nuclei visualization. The endothelial layer was visualized using confocal microscopy.

**RT:qPCR**

Total RNA from cardiac tissue was extracted with TRIzol reagent from Invitrogen Corporation (Carlsbad, CA, USA) following the manufacturer´s instructions. First: strand cDNA was synthesized from 2 μg of total RNA in a 20 μl reaction mixture using the High Capacity cDNA reverse transcription kit, and the qPCR reaction was performed with SYBR select master mix both of Life technologies (Carlsbad, CA, USA). The qPCR conditions were Standard Cycling Mode (Primer Tm ≥60°C) first UDG Activation 50°C 2 min, then AmpliTaq® DNA Polymerase, UP Activation 95°C2 min, denature 95°C 15 sec and anneal/Extend 60°C 1 min.​The following primers were used:

**SNAI1 forward:** 5´ CCA CTG CAA CCG TGC TTT T 3´

**SNAI1 reverse:**5´CAC ATC CGA GTG GGT TTG G 3´

**SNAI2 forward:** 5´CTC ACC TCG GGA GCA TAC AG 5´

**SNAI2 reverse:** 5´GAC TTA CAC GCC CCA AGG ATG 3´

**Actin forward:** 5’ CGA TGC CCT GAG GCT CTT T 3’

**Actin reverse:** 5’ TGG ATG CCA CAG GAT TCC A 3’

**COL1A1 forward:** 5´CTT GGA AAC CTT GTG GAC CAG 3´

**COL1A1 reverse:**5’ TGG CCT TGG AGG AAA CTT TG 3’

**CGTF forward:** 5’TGA CCT GGA GGA AAA CAT TAA GA 3’

**CGTF reverse:** 5´ AGC CCT GTA TGT CTT CAC ACT G 3’

**COL3A1 forward:** 5´ ATTGCCTTGCGTGTTTGATA 3´

**COL3A1 reverse:** 5´AGGCCAGTGGCAATGTAAAG 3´

**MMP2** **forward:** 5’AACTTTGAGAAGGATGGCAAGT 3’

**MMP2** **reverse:** 5’ TGCCACCCATGGTAAACAA 3’

 Target gene expression levels were calculated using the 2: ΔΔCT method with β:actin as the house keeping gene. Results per mouse and target gene were interpreted and visualized relative to mean target gene expression levels in CT mice.

**Immunoblot**

Protein lysates were immunoblotted as described [1]. The immunoreactive bands were visualized with the SuperSignal detection system according to the manufacture's procedures (Pierce, Waltham, MA, USA).

**Immunohistochemistry**

Heart sections were boiled in retrieval buffer for 20 min after xylene deparaffinization. Mouse and Rabbit Specific HRP/DAB (ABC) Detection IHC kit (Abcam) was used by following the manufacturer’s instructions. After incubation overnight at 4°C with the corresponding primary antibodies, the sections were incubated with appropriate secondary antibodies for 1 h at room temperature. Sections were washed and counterstained with Harry´s hematoxylin, dehydrated, and mounted with DPX (Casa Alvarez, Madrid, Spain). Images (1–2 sections, n=5 animals/group) were taken for data quantification using Nikon bright field microscope.

**Confocal microscopy**

Slides containing tissue sections were incubated with the primary antibodies, as described above. After washing with PBS, the slides were incubated with fluorescence -conjugated secondary antibodies, for 1 hour at room temperature. Sections were washed twice with PBS and then mounted with Hoechst. Images were taken for data quantification using a Leyca TCS SP5 confocal microscope (*UAH: NANBIOSIS-CIBER-BNN)*. The fluorescence-conjugated secondary antibodies Alexa 488 Alexa:647, and rabbit anti-mouse-FITC were used. Nuclei were stained with Hoechst. Fluorescent images were captured at × 60 magnification.

**Protein oxidation assay**

Protein oxidation was carried out with the Oxyblot protein detection kit (Chemicon, Temecula, CA, USA) following manufacturer’ instructions. Briefly, total aortic protein lysates (15 μg) were incubated with 2,4-dinitrophenyl hydrazine (DNPH) for 15 min, as recommended by the manufacturer. Proteins were electrophoresed in 15:4% gradient SDS-PAGE gels with 6 μg of derivatized protein/lane. Bands were scanned with an imaging densitometer, and optical densities were quantified.

**miRNA analysis**

miRNeasy Serum/Plasma Advanced Kit from QIAGEN (Hilden, Germany) was used for plasma microRNAs isolation following manufacturer´s instructions. The microRNA eluents were evaluated with the Agilent Bioanalyzer (Santa Clara, CA, USA) and sequenced by the CNIC Genomics Unit. Sequencing reads were pre-processed by means of a pipeline that used FastQC, to asses read quality, and Cutadapt to trim sequencing reads, eliminating Illumina adaptor remains, and to discard those that were shorter than 15 nt or longer than 35 nt after trimming. Resulting reads were mapped against a collection of mature miRNA sequences extracted from miRBase (release 22), to obtain expression estimates with a pipeline that used BWA MEM as aligner and the RSEM function to obtain expression estimates. Expected expression counts were then processed with an analysis pipeline that used Bioconductor package Limma for normalization (using TMM method) and differential expression testing, considering only those miRNA species for which expression was at least 1 count per million (CPM) in 2 or 3 samples. Changes in gene expression are usually considered significant if associated to Benjamini-Hochberg adjusted p-value < 0.05. We have used a 0.2 threshold in the current analyses to increase the number of potentially interesting miRNAs, at the expense of increasing the risk of including false positives. We have used Ingenuity Pathway Analysis (IPA, Qiagen, Hilden, Germany) for functional comparisons between groups, and for figures generation of the enriched canonical pathways.

**References**

1. Cuadrado I, Piedras MJGM, Herruzo I, del Carmen Turpin M, Castejón B, Reventun P, Martin A, Saura M, Zamorano JL, Zaragoza C (2016) EMMPRIN:Targeted magnetic nanoparticles for in vivo visualization and regression of acute myocardial infarction. Theranostics 6:545–557. doi: 10.7150/thno.13352
